# GT-PNID: A lightweight graph-temporal network integrating GCN and transformer for power network intrusion detection

**DOI:** 10.1371/journal.pone.0360854

**Published:** 2026-11-06

**Authors:** Heng Zhou, Mianting Wu, Weixiong Chen, Qiang Zhou

**Affiliations:** 1 The Zhongshan Power Supply Bureau, Guangdong Power Grid Company Limited, Zhongshan, Guangdong, The People’s Republic of China; 2 Shandong University of Technology, Zibo, Shandong, The People’s Republic of China; Beijing Institute of Technology, CHINA

## Abstract

Driven by the advancement of information technology, the transformation of traditional power grids toward intelligence has rendered power systems more vulnerable to cybersecurity risks arising from greater openness and connectivity. Smart grid communication networks exhibit high heterogeneity, diverse protocols, and strict real-time constraints. Existing intrusion detection systems suffer from two key limitations: neglect of spatiotemporal dependencies in network traffic, which hinders the modeling of complex attack evolution, and absence of a unified detection framework with cross-scenario generalization capability. To overcome these limitations, this study develops GT-PNID, a graph-temporal network-based intrusion detection model designed for grid communication networks. The model utilizes a sliding-window dynamic graph construction mechanism to transform heterogeneous traffic into spatiotemporal graph structures. Subsequently, graph convolutional layers are applied to extract local spatial features, while a multilayer Transformer is introduced to model long-range temporal dependencies, followed by graph-level feature fusion for joint spatiotemporal representation. Experiments on CICIDS2017 and NSL-KDD datasets demonstrate that accuracy and F1-scores exceed 99.98%, while the model’s parameter count (424,478) and per-sample inference time (0.09 ms) are substantially lower than mainstream methods, confirming its superior detection capability, lightweight efficiency, and cross-dataset generalization potential for smart grid security.

## 1. Introduction

The evolution of power systems into intelligent grids, termed smart grids, represents a paradigm shift achieved through the deep integration of advanced sensing technologies, communication networks, and intelligent control systems. This paradigm substantially enhances operational efficiency, reliability, and observability compared to conventional power grids. However, the tight coupling of cyber and physical components simultaneously exposes the grid to unprecedented cybersecurity threats. Malicious actors can exploit vulnerabilities within information networks to manipulate measurement data and inject false control commands, potentially enabling lateral movement within operational technology (OT) systems, triggering cascading failures, and even causing widespread power outages [1,2]. Furthermore, advanced false data injection techniques leveraging generative models have been shown to threaten intelligent energy systems [3], underscoring the evolving sophistication of cyber-physical attacks. In recent years, attacks targeting power networks—specifically, the grid communication network examined in this paper—have occurred with increasing frequency. Notable incidents include the 2016 intrusion targeting the Ukrainian power grid, which caused a major blackout [4], as well as subsequent reports of malware infiltration in U.S. power networks [5]. Comparable communication-layer vulnerabilities have been systematically analyzed in smart railway infrastructures [6], highlighting the ubiquity of cybersecurity risks in data communication services across critical sectors. These events sharply underscore the critical vulnerabilities inherent in modern power systems.

In this high-stakes context, intrusion detection systems (IDSs) constitute a vital component of proactive protection strategies for power grid cybersecurity, designed to promptly identify anomalies and halt malicious activities. Existing approaches predominantly fall into two paradigms: conventional machine learning and deep learning. Techniques such as decision trees, random forests, and support vector machines (SVMs) depend on manually engineered features and have demonstrated acceptable performance under specific constraints [7–9]; however, they often struggle to capture the complex and nonlinear interdependencies inherent in network traffic patterns. Deep learning architectures, including convolutional neural networks (CNNs), long short-term memory networks (LSTMs), and recurrent neural networks (RNNs), have yielded marked improvements by automating feature extraction [10–12]. Notably, CNNs demonstrate strong capability in extracting spatial features, whereas sequence models such as LSTMs are adept at modeling temporal dependencies; their combination has produced promising results in prior studies [13,14].

Despite these advances, existing intrusion detection solutions encounter fundamental limitations when deployed in the distinctive operational environment of power grid communication networks.

1) Protocol heterogeneity. Power networks concurrently operate Layer-2 protocols (e.g., IEC 61850 GOOSE/SV for protection) alongside TCP/IP-based protocols (e.g., MMS, DNP3, Modbus TCP), generating fundamentally divergent traffic patterns within a unified infrastructure. This coexistence of disparate communication paradigms renders conventional protocol-specific detection mechanisms inadequate.
2) Extreme latency sensitivity. Protection-grade signals (e.g., GOOSE trip messages) demand sub-millisecond end-to-end latency. As an edge monitoring function that generates intrusion alarms rather than participating in the protection trip path, however, the proposed detector operates within the time budget of supervisory monitoring cycles (e.g., DNP3/IEC 60870-5-104 polling, typically on the order of tens to hundreds of milliseconds), which nonetheless necessitates a lightweight design with minimal computational overhead.
3) Near-zero false-positive tolerance. False alarms in a substation can accidentally trigger unnecessary protective actions or induce operator alarm fatigue across thousands of intelligent electronic devices (IEDs), directly threatening operational reliability.

Compounding these domain-specific barriers, prevailing approaches suffer from two methodological deficiencies: insufficient integration of spatiotemporal dependencies, wherein the complex topological relationships arising from heterogeneous traffic interactions are seldom modeled jointly with temporal attack signatures; and poor cross-scenario generalization, as most detectors are optimized for specific attack categories or datasets, leading to substantial performance degradation in evolving, multi-threat grid environments. Moreover, while graph-temporal networks combining GCN and Transformer have been widely explored in generic intrusion detection, their adaptation to power grid communication networks—particularly regarding dynamic graph construction over heterogeneous protocols and validation on resource-constrained edge platforms—remains insufficient. These intertwined constraints collectively motivate the design of a lightweight, high-precision, and cross-domain generalizable detection framework.

To tackle the above challenges, this study presents GT-PNID (graph-temporal network for power network intrusion detection), a novel detection model tailored for grid communication environments. Distinct from existing graph-temporal approaches that employ static or predefined graph topologies, GT-PNID features a sliding-window dynamic graph construction mechanism guided by feature similarity metrics and an adaptive thresholding strategy, which captures time-varying spatial dependencies among heterogeneous grid traffic flows. GT-PNID synergistically combines graph convolutional networks (GCNs) for modeling spatial dependencies [4,15] with Transformer encoders for capturing long-range temporal patterns via self-attention mechanisms [16,17]. The proposed methodology first transforms raw network traffic sequences into a series of dynamic graph structures. A graph convolutional encoder subsequently extracts the spatial features inherent in each graph, while a multi-layer Transformer encoder models the long-range temporal evolution across the traffic sequence within that graph. The learned spatial and temporal representations are then integrated through a dedicated graph-level fusion mechanism. Beyond benchmark dataset evaluation, the model is further validated on an NVIDIA Jetson TX2 edge platform to confirm its real-time feasibility under substation resource constraints. This integrated approach enables the effective detection of complex, multi-stage attacks targeting grid communication infrastructure.

The main contributions of this study are threefold:

1) GT-PNID, a lightweight, unified spatiotemporal joint detection model integrating GCNs and Transformers and specifically tailored for power grid communication networks, is introduced. While such architectural combinations have been studied in general network security contexts, GT-PNID distinguishes itself through a dedicated dynamic graph construction process and rigorous edge-deployment validation. The presented architecture attains state-of-the-art performance on benchmark intrusion datasets, showing promise for deployment on a resource-constrained edge device in power grid networks while demonstrating strong potential for broader application.
2) A dynamic graph construction method based on sliding windows and adaptive similarity metrics with percentile-based thresholding is developed. Unlike static graph representations used in prior graph-temporal intrusion detection systems, this approach constructs distinct graph structures for each temporal window, effectively transforms sequential network traffic flows into temporal graph sequences, adapting to the spatiotemporal dynamics inherent in grid communication network traffic and improving the capture of both spatial correlations among traffic flows and the temporal evolution of attack signatures.
3) A comprehensive empirical evaluation is conducted on two public benchmark datasets (CICIDS2017 and NSL-KDD) and on a resource-constrained edge platform (NVIDIA Jetson TX2). GT-PNID achieves accuracy and F1-scores over 99.98% on both datasets, exceeding current state-of-the-art methods and demonstrating robust detection capability, promising generalization across distinct data distributions, lightweight efficiency (only 424,478 parameters and 0.09 ms per-sample inference), and consistent performance across diverse network attack scenarios.

The structure of this paper is as follows: Section 2 surveys the existing literature. Section 3 details the GT-PNID architecture, encompassing model formulation and the overall framework. Section 4 describes the detailed implementation of the GT-PNID model. Section 5 presents the experiments and discusses the results. Section 6 summarizes the key findings and discusses potential directions for subsequent work.

## 2. Related works

### 2.1. Intrusion detection using classical machine learning

In the field of grid communication network security, intrusion detection systems employing traditional machine learning (ML) methods have been extensively investigated. Du et al. [18] presented a thorough review of cybersecurity analysis, attack detection, and defense mechanisms in cyber-physical power systems, examining both state estimation-based and ML-based detection approaches. Early research predominantly utilized single ML algorithms for attack detection. Stryczek and Natkaniec [9] applied SVM methods to analyze smart grid network traffic, validating the effectiveness of SVM in grid environments. Havlena et al. [10] developed an effective automata-based detection method for network attacks in smart grid communication, in which normal communication protocol behavior was precisely modeled, enabling high-precision anomaly detection with low false-positive rates. Alani et al. [8] proposed a two-stage intrusion detection and classification framework for smart grids, where feature engineering was combined with decision tree classifiers to effectively identify multiple types of network threats. Yuan et al. [19] compared the performance of k-means clustering and autoencoders in power system attack detection, and the results demonstrated that traditional clustering methods possess certain detection capabilities in simple attack scenarios.

As research progressed, ensemble learning and federated learning methods were introduced to further enhance detection performance. Alsirhani et al. [20] proposed an ensemble learning-driven intrusion detection framework tailored to smart grids, constructing an ensemble model by combining logistic regression, random forest, and k-nearest neighbors, and validated its effectiveness on two benchmark datasets. Idowu et al. [21] systematically evaluated the effectiveness of ML techniques in distributed DoS attack detection, noting that ensemble methods such as random forest perform excellently when handling large-scale feature sets. Jithish et al. [22] explored federated learning for distributed intrusion detection across smart grid nodes, where local models were trained on individual nodes and aggregated updates were performed, achieving collaborative detection while preserving data privacy. Mazhar et al. [23] conducted an analysis of cyber-attacks and defense mechanisms for smart grids using ML and blockchain methods, discussing the applicability of different ML algorithms in grid security protection. Nevertheless, traditional ML methods depend on handcrafted feature design, struggling to model the complex nonlinear patterns inherent in network traffic, while spatial dependencies among grid components and the temporal dynamics of attack behaviors remain largely neglected.

### 2.2. Intrusion detection using deep learning

The continuous evolution of deep learning has significantly propelled the field of intrusion detection in grid communication networks. Various deep neural network (DNN) architectures have been leveraged for intrusion identification within smart grid communication networks.

In the deployment of CNNs, Diaba et al. [11] proposed a CNN-based DDoS detection algorithm for smart grids, where convolutional layers were employed to extract spatial features from network traffic, enabling efficient identification of DDoS attacks. In the deployment of RNNs and LSTMs, Kasongo [12] proposed an RNN-based intrusion detection framework, where recurrent structures were utilized to capture temporal dependencies in sequential data. Yuan et al. [13] employed LSTM for real-time event identification based on PMU data, validating the effectiveness of temporal models in dynamic monitoring of power systems. Amutha et al. [14] investigated LSTM-based anomaly detection and classification methods for PMU streaming data, achieving favorable results in mining temporal patterns from synchrophasor measurement data.

To further integrate spatial and temporal features, Alsaiari et al. [24] proposed a hybrid CNN-LSTM framework for smart grid intrusion detection, where local spatial features were first extracted using CNN layers and temporal dependencies were subsequently captured through LSTM layers, outperforming single-architecture models and validating the effectiveness of spatiotemporal feature fusion. In addition, other fundamental deep learning approaches have been explored. Abdelkhalek et al. [25] developed a DNN-based anomaly detection architecture for distributed energy resource communication, designing a dedicated detection architecture tailored to DER communication scenarios. Berghout et al. [26] proposed a robust data engineering approach for PMU adversarial attacks, combining autoencoders with DNNs to enhance model defense capabilities against malicious attacks. Foggo et al. [27] introduced the pmuBAGE benchmark dataset for power system events, providing standardized data support for the development and assessment of deep learning-driven detection methods. Anitha [28] developed a Bi-LSTM-CNN fused malicious traffic detection method, enhancing temporal modeling capability through bidirectional LSTM and further improving detection performance.

Recently, graph neural networks (GNNs) have exhibited distinct strengths in processing graph-structured data, enabling effective modeling of relational dependencies among nodes. This characteristic renders them well-suited for intrusion detection in grid communication networks, where communication entities and their interactions can be naturally represented as graph structures. Sweeten et al. [4] developed a GNN-based multimodal intrusion detection system that fuses cyber-physical features, achieving detection rates up to 16% higher than those obtained with single-modal inputs. Jiang et al. [15] proposed a false data injection attack detection method combining graph attention networks with spatiotemporal features, where the weight relationships among different nodes were adaptively learned using GAT, enabling effective identification of stealthy attacks.

To address the gradient vanishing problem encountered by RNNs and LSTMs when modeling long sequences, the Transformer model leverages its self-attention mechanism to capture long-range temporal dependencies in parallel. This capability enables more efficient modeling of temporal patterns in network traffic. Lakshmanan et al. [16] proposed a GNN and Transformer encoder framework for intrusion detection in smart grids, where GNN was employed to extract spatial features from the power system topology, and the Transformer encoder was used to capture long-range dependencies. Yuan and Li [17] proposed the STSA-Transformer algorithm for APT detection in power systems, where local features were extracted through spatial-temporal encoding and global features of traffic sequences were captured using the Transformer encoder, outperforming traditional deep learning approach in both detection accuracy and false-positive rates. Ullah et al. [29] proposed TNN-IDS, a Transformer and neural network integrated intrusion detection system for IoT networks, validating the effectiveness of the attention mechanism in identifying network threats. Jafari et al. [30] proposed an attention mechanism- driven deep learning approach to enhance the cybersecurity protection capability of transformer differential relays, where sequential patterns in current measurement data were effectively captured using the self-attention mechanism of Transformers, achieving excellent attack detection accuracy.

Based on the literature reviewed above, current intrusion detection systems are predominantly designed for specific attack types or particular network environments, lacking a unified detection framework with cross-scenario generalization capability. Furthermore, many studies neglect spatiotemporal feature fusion when constructing detection systems. To address these limitations, this paper proposes GT-PNID, a grid communication network intrusion detection model that integrates GCNs with Transformers.

## 3. GT-PNID architecture

### 3.1. Model formulation

The model proposed in this work synergistically combines the representational strengths of graph convolutional architectures and Transformer-based sequence modeling. Its design features a dynamic graph construction process and a dedicated spatiotemporal feature fusion strategy, which jointly enable effective and precise detection of advanced network intrusion attacks targeting grid communication infrastructure.

Mathematically, the input to the model is defined as a set of heterogeneous traffic flows from grid communication networks, denoted as $S=\{\left({\text{A}}^{\left(i\right)},c^{\left(i\right)}\right){\}}_{i=\text{1}}^{N}$. Here, N denotes the total number of samples in the dataset. For each sample i, the feature vector is ${\text{A}}^{\left(i\right)}\in R^{\rho }$, where ρ represents the feature dimensionality. The corresponding label vector $c^{\left(i\right)}$ takes values in {1,2,…,C}, with C being the total number of intrusion categories.

A sliding window mechanism is employed to segment raw sequential network traffic data so as to capture its spatiotemporal evolution, thereby converting the data into a sequence of dynamic graphs. Given a predefined window length ω and stride δ, consecutive windows of size ω yield local feature matrices:


$$\begin{array}{c} \begin{array}{c} {𝐀}_{u}=[\begin{array}{c} {\text{A}}^{\left(u\delta +\text{1}\right)}\\ {\text{A}}^{\left(u\delta +\text{2}\right)}\\ \vdots \\ {\text{A}}^{\left(u\delta +\omega \right)} \end{array}]\in R^{\omega \times \rho } \end{array} \end{array}$$ (1)

where u∈{0,1,…,T−1} is the window index, T=⌊(N−ω)/δ⌋+1 is the total number of windows, and the semicolon denotes vertical concatenation. The corresponding label set for window u is ${𝐜}_{u}=\left[c^{\left(u\delta +\text{1}\right)},c^{\left(u\delta +\text{2}\right)},\ldots ,c^{\left(u\delta +\omega \right)}\right]$.

This process yields a series of graph structures $\text{G}=\{G_{\text{1}},G_{\text{2}},\ldots ,G_{T}\}$. A graph at time window u is defined as $G_{u}=\left(V_{u},E_{u},\Gamma_{u},c_{u}\right)$, containing its vertex set $V_{u}$, edge set $E_{u}$, an edge attribute matrix $\Gamma_{u}$, and a corresponding graph-level label $c_{u}$. Here, $V_{u}=\{\text{1},\text{2},\ldots ,\omega \}$ denotes the vertex set comprising ω nodes, each corresponding to a traffic sample in window u and associated with the feature vector ${\text{A}}_{u}^{\left(\alpha \right)}={\text{A}}^{\left(u\delta +\alpha \right)}$ for α∈{1,…,ω}.

It should be noted that the graph constructed here is a traffic-sample similarity graph rather than a representation of the physical or communication topology of a power grid: each node corresponds to an individual traffic record within the sliding window, and edges encode feature-level similarity rather than device-to-device connectivity. The specific similarity metric is formally defined in Section 4.1. Nevertheless, traffic samples generated by the same type of device (e.g., RTUs or IEDs) or belonging to the same communication session tend to exhibit similar feature vectors; consequently, this similarity graph can be interpreted as an implicit, data-driven approximation of device behavioral grouping. The dynamic edge weights thereby capture evolving behavioral correlations among semantically related flows, which may indicate coordinated attacks or device-group anomalies.

The primary objective of the model is to learn a mapping function $F:G_{u}\mapsto {\hat{c}}_{u}$ that predicts the graph-level label from the corresponding graph snapshot. This learning process is accomplished by optimizing the model parameters through minimization of the standard cross-entropy loss, ultimately yielding accurate multi-class classification of diverse intrusion activities.

### 3.2. Model framework

The architecture of the proposed approach, depicted in Fig 1, comprises four principal modules: 1) data preprocessing, 2) graph construction, 3) spatiotemporal feature encoding, and 4) final classification.

**Fig 1:**
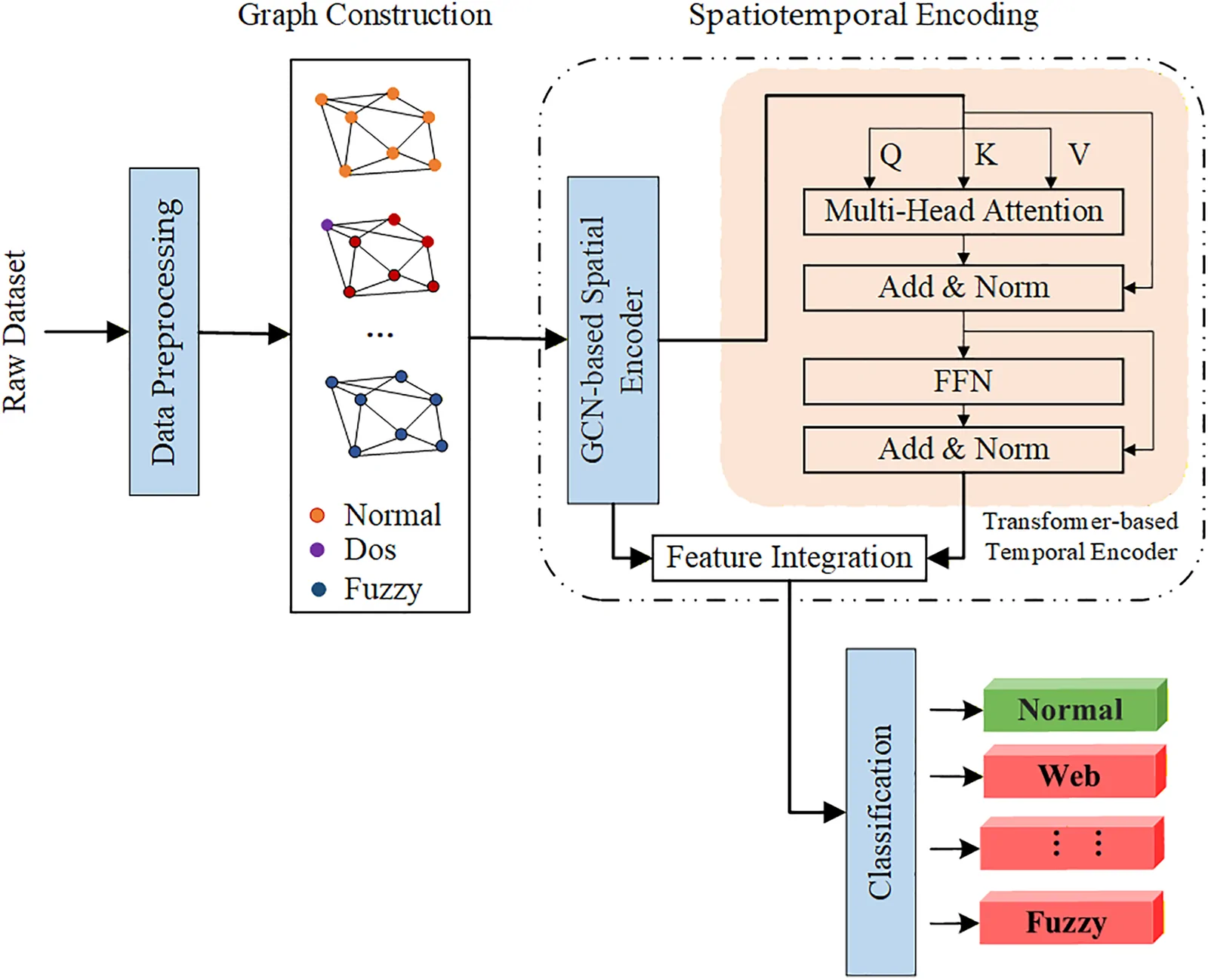
Schematic overview of the proposed GT-PNID framework.

The data preprocessing stage ensures data quality by addressing missing values, detecting outliers, normalizing features, and mitigating class imbalance, thereby producing a clean and reliable input stream. Subsequently, the graph construction module employs a sliding window over the preprocessed data sequence to generate a temporal succession of graph snapshots. The edges within each graph are established based on similarity measures computed between node features.

The spatiotemporal encoding module, which constitutes the core of this framework, comprises two synergistic components: a graph convolutional encoder responsible for capturing the relational structures and local spatial dependencies among nodes, and a Transformer-based temporal encoder that models long-range sequential dependencies across the sample sequence within each sliding window. Finally, a dedicated classifier layer processes the fused spatiotemporal representation to generate the ultimate intrusion category prediction.

## 4. Implementation of the proposed GT-PNID model

### 4.1. Dynamic graph construction

To effectively capture the dynamically evolving feature correlations inherent in grid communication network traffic, a flexible dynamic graph construction method is introduced. Conventional static graph representations are fundamentally limited in their capacity to model such time-varying dependencies. The proposed approach overcomes this limitation by constructing distinct graph structures for each discrete temporal window, guided by a combination of feature similarity metrics and an adaptive thresholding strategy.

The input spatiotemporal traffic data are first segmented using a sliding window of size ω. For each window u, the local feature matrix ${𝐀}_{u}$ and its corresponding label set ${𝐜}_{u}$ are extracted. Cosine similarity is then adopted as the adjacency measure, and pairwise similarity values are computed for all samples within the window to construct the similarity matrix $\Lambda \in R^{\omega \times \omega }$, defined as follows:


$$\begin{array}{c} \begin{array}{c} \Lambda^{\alpha \beta }=\frac{{𝐀}_{u}^{\left(\alpha \right)}\cdot {𝐀}_{u}^{\left(\beta \right)}}{∥{𝐀}_{u}^{\left(\alpha \right)}{∥}_{\text{2}}∥{𝐀}_{u}^{\left(\beta \right)}{∥}_{\text{2}}+\eta },\;\forall \alpha ,\beta \in [\omega ] \end{array} \end{array}$$ (2)

where ${𝐀}_{u}^{\left(\alpha \right)}$ and ${𝐀}_{u}^{\left(\beta \right)}$ denote the feature vectors of sample α and sample β within the current window u, respectively. A small constant η (e.g., ${\text{1}\text{0}}^{-\text{8}}$) is introduced in the denominator to ensure numerical stability.

After excluding self-similarity values from the similarity matrix, a dynamic threshold θ is derived as the π-th percentile of the remaining similarity scores. This percentile-based, window-adaptive thresholding mechanism serves to preserve consistent graph connectivity across consecutive temporal windows, thereby facilitating the training stability of subsequent graph-based neural networks.

For each sample, only the top-κ non-self neighbors with the highest similarity scores are retained. An edge between two distinct samples α and β is established exclusively when their similarity $\Lambda^{\alpha \beta }$ exceeds the threshold θ, with the similarity value assigned as the corresponding edge weight. This dual-criterion mechanism—percentile-based thresholding combined with top-κ sparsification—balances graph sparsity and the preservation of key correlations.

The graph-level label $c_{u}$ for each temporal window is determined by aggregating the labels of its constituent traffic samples. Specifically, if all samples within a given window correspond to normal traffic, the graph is assigned the label “normal”. Otherwise, the graph inherits the label of the most frequently occurring attack type within that window. Formally, given ${𝐜}_{u}=\left[c^{\left(\text{1}\right)},c^{\left(\text{2}\right)},\ldots ,c^{\left(\omega \right)}\right]$ as the set of sample labels within windowu, the graph-level label $c_{u}$ is computed as


$$\begin{array}{c} \begin{array}{c} c_{u}=\left\{ \begin{array}{cc} @ll"\text{normal}", & \forall c\in {𝐜}_{u},c="\text{normal}"\\ \text{mode}(\{c\in {𝐜}_{u}|c\neq "\text{normal}"\}), & \text{otherwise} \end{array}\right. \end{array} \end{array}$$ (3)

Through this procedure, the original sequential network traffic data are systematically transformed into a sequence of dynamic graphs: $\text{G}=\{\left(V_{u},E_{u},\Gamma_{u},c_{u}\right){\}}_{u=\text{1}}^{T}$, where T is the total number of temporal windows. The node feature matrix ${𝐀}_{u}\in R^{\omega \times \rho }$ is associated with $V_{u}$ as node attributes. This graph sequence constitutes the primary input to the spatiotemporal encoder, allowing the model to explicitly capture and exploit the time-evolving spatial correlations embedded in the data. It should be noted that the majority-vote labeling in (3) assumes temporal locality of attack behaviors within each window. While this assumption largely holds for the continuous attack flows prevalent in the evaluated datasets, simultaneous mixed attacks within a single window could introduce label ambiguity by diluting minority-attack signatures. In practice, this effect can be mitigated by tuning the window stride and size to balance label purity and temporal context; future work will further investigate node-level multi-label classification or hierarchical graph labels to resolve such ambiguity without resorting to window-level aggregation.

The detailed procedure is presented in Algorithm 1:

**Algorithm 1.** Dynamic Graph Construction.

**Input:** Raw traffic feature matrix $𝐀\in R^{N\times \rho }$; label vector $𝐜\in R^{N}$; sliding window size ω; stride δ; neighbor limit κ; similarity percentileπ

**Output:** Graph sequence: $\text{G}=\{G_{u}\}$ with $G_{u}=\left(V_{u},E_{u},\Gamma_{u},c_{u}\right)$, where $V_{u}$: vertex set, $E_{u}$: edge set, $\Gamma_{u}$: edge weights, $c_{u}$: graph label

1. Initialize𝔊←∅

2. Compute total windows:T←⌊(N−ω)/δ⌋+1

3. for u=0 to T−1 do

4. start←u·δ

5. Extract ${𝐀}_{u}\leftarrow 𝐀\left[\text{start}:\text{start}+\omega \right]$,${𝐜}_{u}\leftarrow 𝐜\left[\text{start}:\text{start}+\omega \right]$

6. Initialize vertex set$V_{u}\leftarrow \{\text{0},\text{1},\ldots ,\omega -\text{1}\}$

7. Compute similarity matrix Λ using (2)

8. Let$\Psi \leftarrow \{\Lambda^{\alpha \beta }|\alpha \neq \beta \}$

9. Set dynamic threshold θ←percentile(Ψ,π)

10. Initialize $E_{u}\leftarrow \emptyset$,$\Gamma_{u}\leftarrow \emptyset$

11.   for α=0 to ω−1 do

12.    $C_{\alpha }\leftarrow \{\beta |\beta \neq \alpha \wedge \Lambda^{\alpha \beta }>\theta \}$

13.    $N_{\alpha }\leftarrow \text{top}-\kappa \left(C_{\alpha },\Lambda^{\alpha \beta }\right)$// If $\left|C_{\alpha }\right|<\kappa$, take all

14.     for each $\beta \in N_{\alpha }$ do

15.     if $(\beta ,\alpha )\notin E_{u}$ then// Avoid duplicate

16.        Add edge (α,β) to $E_{u}$, add weight $\Lambda^{\alpha \beta }$ to$\Gamma_{u}$

17.     end if

18.    end for

19.   end for

20. Set graph label $c_{u}$ using (3)

21. Create$G_{u}\leftarrow \left(V_{u},E_{u},\Gamma_{u},c_{u}\right)$

22. Append $G_{u}$ to𝔊

23. end for

24. returnG

It should be specifically noted that this similarity graph is induced from traffic-feature cosine similarity rather than from the physical grid topology; its relevance to power-grid intrusion detection stems from the tendency of samples sharing the same device type or communication session to cluster together.

### 4.2. Spatiotemporal encoding

The spatiotemporal encoding module constitutes the core feature learning unit of the GT-PNID framework. Its primary objective is to extract discriminative representations by jointly modeling the intrinsic spatial relationships and temporal dynamics inherent in network traffic. As shown in Fig 1, this is accomplished through three tightly integrated components: a graph convolutional encoder that aggregates neighborhood features to capture local spatial dependencies, a Transformer-based sequential encoder that models long-range temporal dependencies among the ω consecutive traffic samples within each dynamic graph snapshot, and a feature integration sub-module that unifies the spatial and temporal feature representations. Together, these components yield a unified representation encoding both spatial and temporal patterns.

#### 4.2.1. GCN-based spatial encoder.

The GCN-based spatial encoder serves as the primary mechanism for learning representations from graph-structured inputs. Fig 2 depicts the architecture of the encoder, which comprises two stacked GCN layers. The first layer processes the node features ${𝐀}_{u}$ associated with vertex set $V_{u}$ to learn representations enriched with first-order spatial neighborhood information. The resulting representation is then fed into the subsequent GCN layer, whose deeper receptive field captures higher-order structural relationships among nodes. Both layers employ the ReLU nonlinearity and incorporate Dropout regularization to improve representation capacity and mitigate overfitting. Formally, the layer-wise propagation mechanism is formulated as:

**Fig 2:**
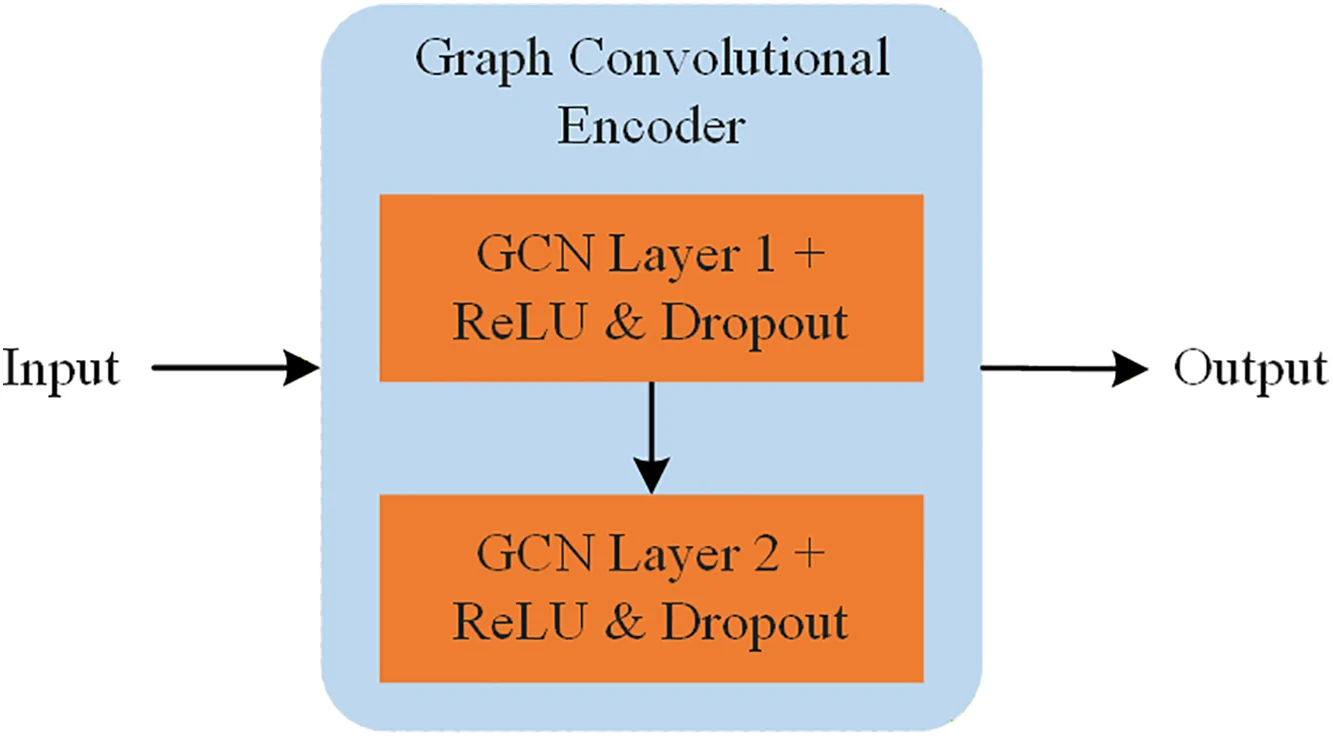
GCN-based spatial encoder.


$$\begin{array}{c} \begin{array}{c} {𝐇}^{\left[l+\text{1}\right]}=\varphi \left({\tilde{\Delta }}^{-\frac{\text{1}}{\text{2}}}\tilde{𝐁}{\tilde{\Delta }}^{-\frac{\text{1}}{\text{2}}}{𝐇}^{\left[l\right]}\Phi^{\left[l\right]}\right) \end{array} \end{array}$$ (4)

where:

${𝐇}^{\left[l\right]}$ is the activation matrix at layer [l], and ${𝐇}^{\left[\text{0}\right]}={𝐀}_{u}$ is the initial activation matrix.

$\Phi^{\left[l\right]}$ denotes a layer-specific learnable weight matrix;

$\tilde{𝐁}=𝐁+𝐈$ is the adjacency matrix augmented with self-loops;

𝐈 is the unit matrix;

$\tilde{\Delta }$ denotes the degree matrix of $\tilde{𝐁}$;

φ(·) is the nonlinear transformation function.

This design produces refined node embeddings ${𝐇}_{g}\in R^{B\times w\times d_{g}}$ that effectively encode the local graph topology and spatial dependencies, where B denotes the batch size.

#### 4.2.2. Transformer-based temporal encoder.

Fig 3 presents an overview of the temporal encoder module, which adopts a stacked Transformer architecture to model the temporal dependencies among the ω traffic samples within each window. The input to this encoder is the graph-structured representation ${𝐇}_{g}$ produced by the preceding GCN encoder. To encode the temporal order of the graphs along the time dimension, fixed sinusoidal positional encodings Ξ are injected into the input embeddings, yielding the enhanced representation ${𝐇}_{PE}={𝐇}_{g}+\Xi$. This positional encoding Ξ for a given position μ∈{1,…,ω} (the temporal index of a traffic sample within the window) and feature dimension ν is defined as:

**Fig 3:**
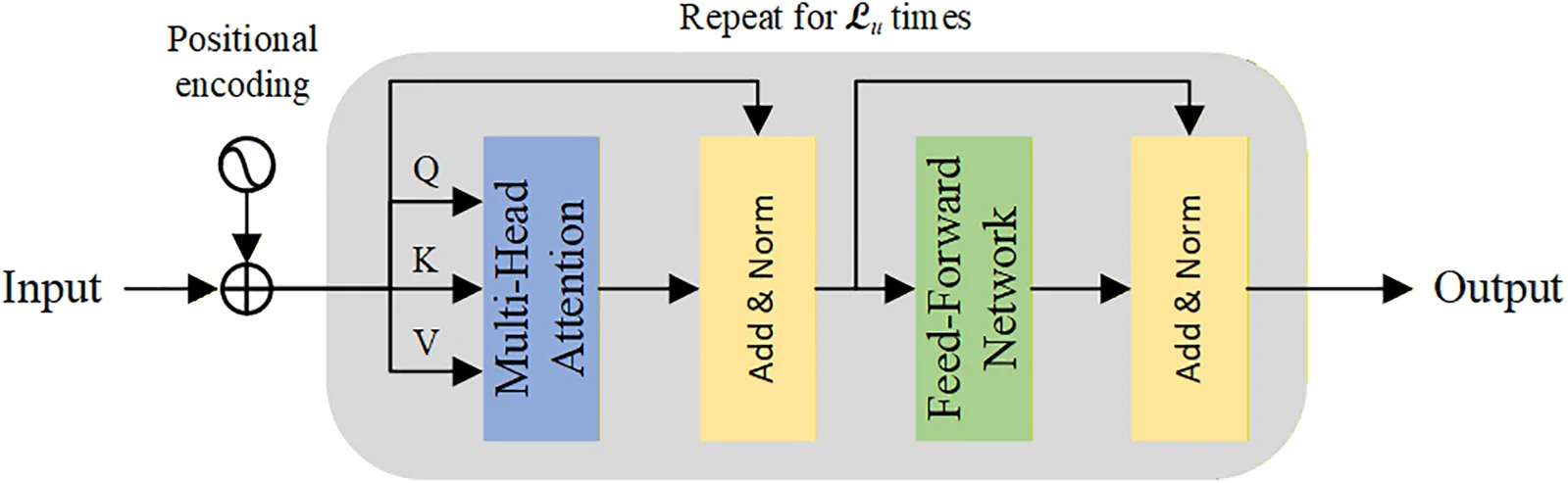
Transformer-based temporal encoder.


$$\begin{array}{c} \begin{array}{c} \Xi_{\left(\mu ,\nu \right)}=\left\{ \begin{array}{cc} \text{\textbackslash{}!\textbackslash{}!\textbackslash{}!sin}\left(\frac{\mu }{{\text{1}\text{0}\text{0}\text{0}\text{0}}^{\frac{\nu }{d_{g}}}}\right), & \nu \text{ is even}\\ {}[9pt]\text{cos}\left(\frac{\mu }{{\text{1}\text{0}\text{0}\text{0}\text{0}}^{\frac{\nu -\text{1}}{d_{g}}}}\right), & \nu \text{ is odd} \end{array}\right. \end{array} \end{array}$$ (5)

The core of each Transformer encoder layer is the self-attention mechanism, which evaluates pairwise relevance over the sequence of ω intra-window samples to capture their long-range temporal dependencies. The process begins by projecting the input $H_{PE}$ into query vectors $Q=H_{PE}\cdot W_{Q}$, key vectors $K=H_{PE}\cdot W_{K}$, and value vectors $V=H_{PE}\cdot W_{V}$, where $W_{Q},W_{Q},W_{V}$ are trainable projection matrices. The scaled dot-product attention is then calculated:


$$\begin{array}{c} \begin{array}{c} Attn(Q,K,V)=Softmax\left(\frac{QK^{T}}{\sqrt{d_{k}}}\right)V \end{array} \end{array}$$ (6)

where $d_{k}$ denotes the key vector dimensionality and the division by $\sqrt{d_{k}}$ serves as a scaling factor to stabilize gradient flow.

The remaining components of the Transformer layer—including multi-head self-attention, layer normalization, residual connections, and position-wise feed-forward networks—follow the standard architecture [31]. Collectively, a single Transformer layer performs the following mapping:


$$\begin{array}{c} \begin{array}{c} {𝐇}_{u}=T_{\varphi }\left({𝐇}_{PE}\right) \end{array} \end{array}$$ (7)

where $T_{\varphi }(\cdot )$ denotes the composite transformation parameterized by φ. After $L_{u}$ stacked layers, the final temporal feature representation ${𝐇}_{u}\in R^{B\times \omega \times d_{u}}$ is obtained, which encapsulates the long-range dynamic patterns of the sample sequence within the window.

#### 4.2.3. Feature integration.

The feature integration module, as depicted in Fig 4, unifies the spatial and temporal feature representations derived from the graph convolutional and temporal encoders.

**Fig 4:**
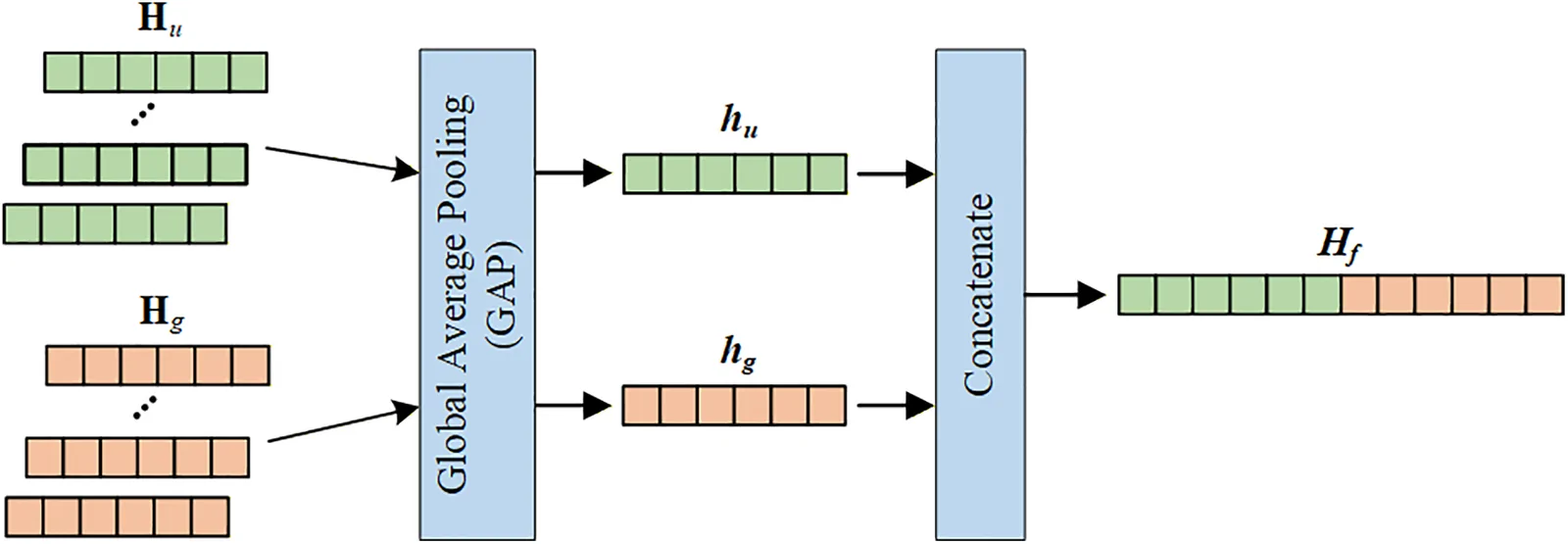
Process of feature integration.

The feature integration sub-module, as depicted in Fig 4, unifies the spatial and temporal feature representations derived from the spatial and temporal encoders.

First, each branch of the encoder produces high-dimensional node-level feature maps (${𝐇}_{g}$ from the GCN and ${𝐇}_{u}$ from the Transformer). To distill these into informative global representations, global average pooling (GAP) is applied along the time-step dimension. This operation mean-pools each node’s features across the ω time steps within the window, yielding two compact global feature vectors:


$$\begin{array}{c} \begin{array}{c} h_{g}=GAP\left({𝐇}_{g}\right),h_{u}=GAP\left({𝐇}_{u}\right) \end{array} \end{array}$$ (8)

where $h_{g}\in R^{B\times d_{g}}$, $h_{u}\in R^{B\times d_{u}}$.

$h_{g}$ captures the aggregated spatial-topological information about the graph structure, while $h_{u}$ distills the long-range temporal dependencies across the ω consecutive traffic samples within the window into a fixed-length vector. These pooled vectors are then fused along the feature dimension to yield the final integrated embedding:


$$\begin{array}{c} \begin{array}{c} H_{f}=Concat(h_{g},h_{u}) \end{array} \end{array}$$ (9)

where $H_{f}\in R^{B\times (d_{g}+d_{u})}$.

This fusion step is critical, as it generates a comprehensive feature vector $H_{f}$ that jointly embodies the structural information encoded by the GCN and the evolving temporal dynamics captured by the Transformer. This fused vector constitutes the unified input to the downstream classification stage, allowing the model to make predictions based on a combined representation of both spatial and temporal data characteristics.

### 4.3. Classification

The classification module processes the fused spatiotemporal feature vector $H_{f}$ to produce the final intrusion detection predictions. As illustrated in Fig 5, this module consists of a fully connected network (FCN) activated by ReLU, with subsequent Dropout regularization and a Softmax classifier, collectively mapping the fused representation to class probability distributions.

**Fig 5:**
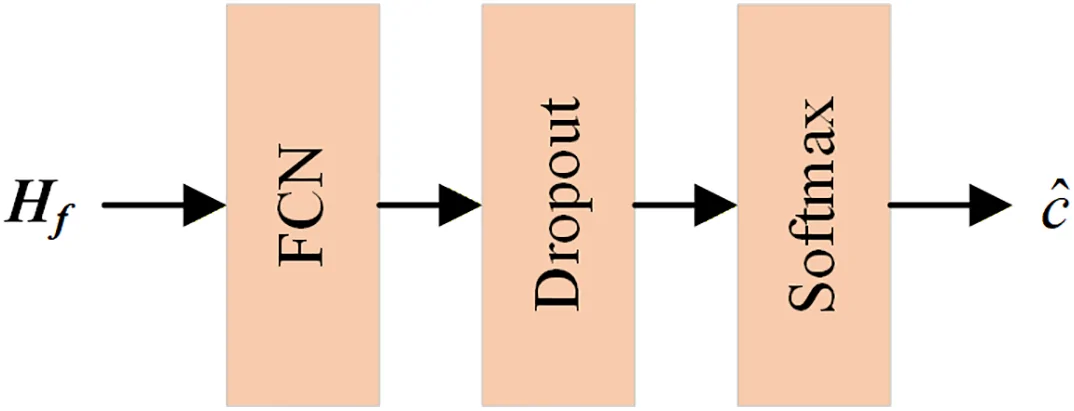
Structure of the classification module.

The final prediction is assigned to the class with the highest probability score. To optimize the model parameters, the multi-class cross-entropy loss serves as the optimization objective during training. For a batch of size B comprising graph-structured samples, the ground-truth labels are denoted by $C=\{c_{\text{1}},c_{\text{2}},\ldots ,c_{B}\}$, where $c_{b}\in R^{C}$ is a one-hot encoded vector representing the graph-level label (as defined in (3)) of the b-th graph sample in the batch. The predicted probability distributions are represented as $\hat{C}=\{{\hat{c}}_{\text{1}},{\hat{c}}_{\text{2}},\ldots ,{\hat{c}}_{B}\}$, with ${\hat{c}}_{b}\in R^{C}$. The objective function is formulated as:


$$\begin{array}{c} \begin{array}{c} J=-\frac{\text{1}}{B}\sum_{b=\text{1}}^{B}\sum_{\gamma =\text{1}}^{C}c_{b}^{\gamma }\text{log}\left({\hat{c}}_{b}^{\gamma }\right) \end{array} \end{array}$$ (10)

where $c_{b}^{\gamma }$ and ${\hat{c}}_{b}^{\gamma }$ correspond to the ground-truth and predicted probability values that the b-th graph sample belongs to class γ, respectively. Although the dynamic graph construction module (Section 4.1) employs non-parametric operations—cosine similarity, percentile-based thresholding, and top-κ neighbor selection—with no trainable parameters, the downstream neural network pipeline is trained end-to-end. The neural network components learn to map the dynamically constructed graph structures to classification outputs, and their parameters (from the graph encoder to the final classification layer) are optimized via backpropagation using the loss function defined in (10), thereby improving the model’s discriminative capability for distinguishing benign traffic from diverse intrusion patterns.

## 5. Experiments and results

### 5.1. Experimental configuration

This section details the computational environment, evaluation datasets, performance metrics, and model optimization procedures adopted in this study.

#### 5.1.1. Computational environment and datasets.

The proposed model was built and optimized with the PyTorch deep learning framework. All training and evaluation were executed on a machine configured with an Intel Core i5-12600KF processor, an NVIDIA GeForce RTX 4060 graphics card, and 32 GB of memory. To ensure statistical reliability, all experiments are repeated using a predefined set of random seeds, and the reported metrics represent the mean values across these runs.

Although real-world power network traffic data with labeled attacks remain scarce owing to security and privacy constraints, the selected benchmark datasets (CICIDS2017 and NSL-KDD) provide rigorous testbeds for evaluating the model’s core capabilities, notwithstanding their lack of power-specific protocol semantics (e.g., IEC 61850 GOOSE/SV, MMS, DNP3, IEC 60870-5-104), for the following reasons. First, the attack categories represented in these datasets—including DoS, DDoS, port scanning, and brute-force attacks—constitute the most prevalent cyber threats against power SCADA and control systems; a DDoS attack targeting a front-end processor, for instance, can effectively blind grid operators to real-time system conditions. Second, achieving strong performance across two datasets with distinct feature distributions serves to validate the model’s cross-domain generalizability, a critical requirement for heterogeneous power sub-networks spanning generation, transmission, and distribution environments. Third, public benchmarks enable standardized evaluation of computational efficiency, allowing direct assessment of whether the model satisfies the sub-millisecond inference latency and lightweight resource footprint demanded by substation edge deployment scenarios. Nevertheless, we recognize that validation on these generic datasets is a necessary but not sufficient step; future work must extend to real or high-fidelity simulated power network traffic containing actual grid protocols and labeled attacks (e.g., protocol-level message spoofing), as explicitly discussed in Section 6.

Accordingly, to comprehensively evaluate intrusion detection performance in the context of grid communication networks, experiments were carried out on these two benchmark datasets:

- The NSL-KDD dataset is a refined derivative of the classic KDDCup’99. Its key advantage lies in the elimination of redundant records and a more balanced sample distribution across traffic categories, encompassing normal traffic along with four primary attack families (DoS, Probe, R2L, and U2R). Each sample is characterized by 41 distinct features. Owing to its inherently reasonable class balance, no explicit rebalancing preprocessing was applied for experiments involving this dataset.
- The CICIDS2017 dataset is a comprehensive and widely-used contemporary benchmark that encapsulates a diverse set of modern attack profiles, including DDoS attacks and port scanning. It provides rich, 78-dimensional feature vectors for traffic characterization. To address the pronounced intrinsic class imbalance, SMOTE is first applied to augment minority attack classes, followed by controlled random undersampling of the majority normal class. Both operations are strictly confined to the training partition, applied after the chronological train–test split but prior to sliding-window construction. Each synthetic sample is temporally anchored via an interpolated timestamp derived from its two parent samples and inserted in chronological order; undersampling strictly preserves the temporal order of all retained records, as detailed in Algorithm 2. We note that synthetic records do not represent real traffic measurements, such that the resampled training sequence approximates the temporal evolution pattern of the original traffic. This approximation, however, is exclusively limited to the training phase, as the test partition is never subjected to resampling, imputation, or reordering. The statistical distributions of the prepared datasets are visualized in Figs 6 and 7.

**Fig 6:**
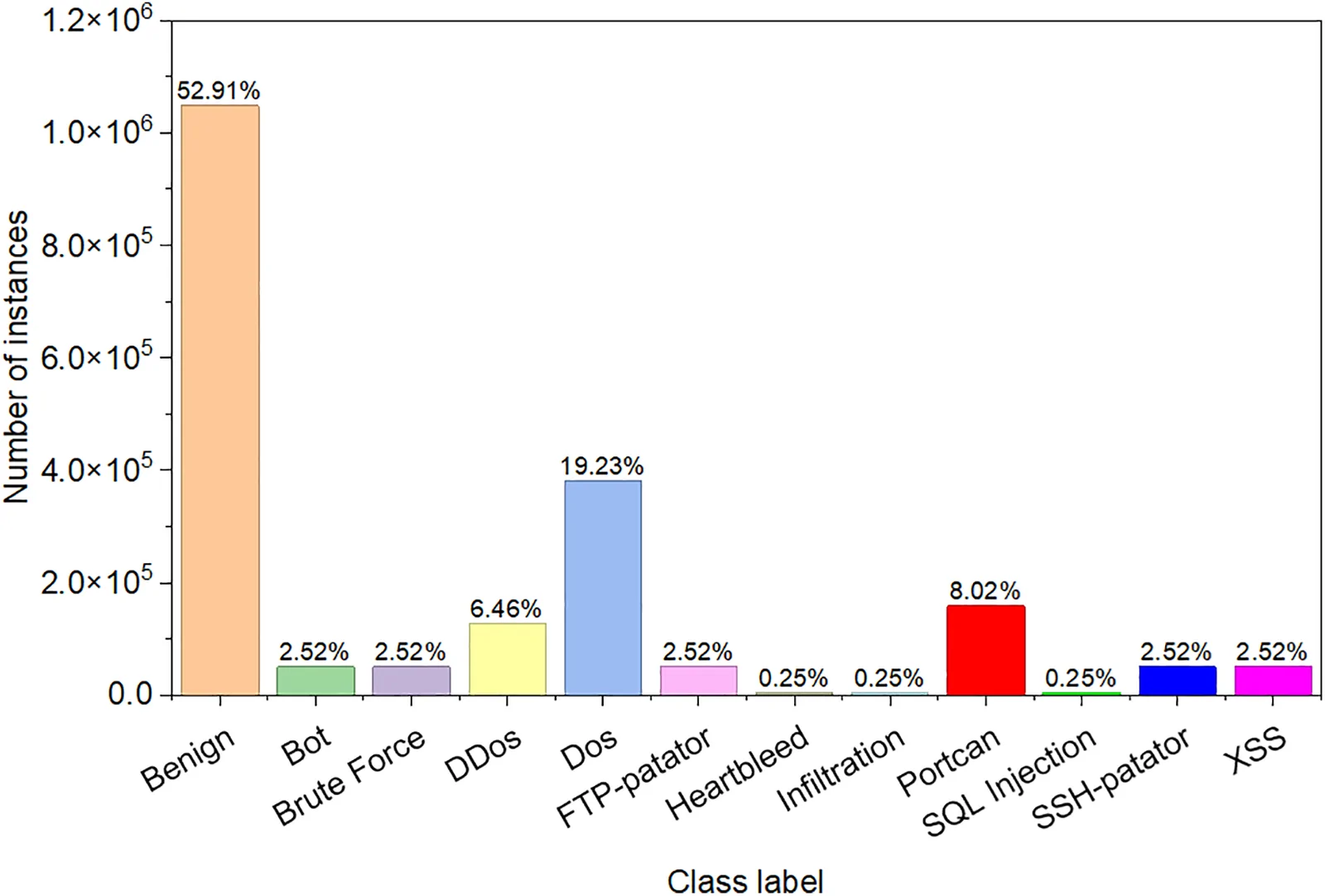
CICIDS2017 dataset statistics.

**Fig 7:**
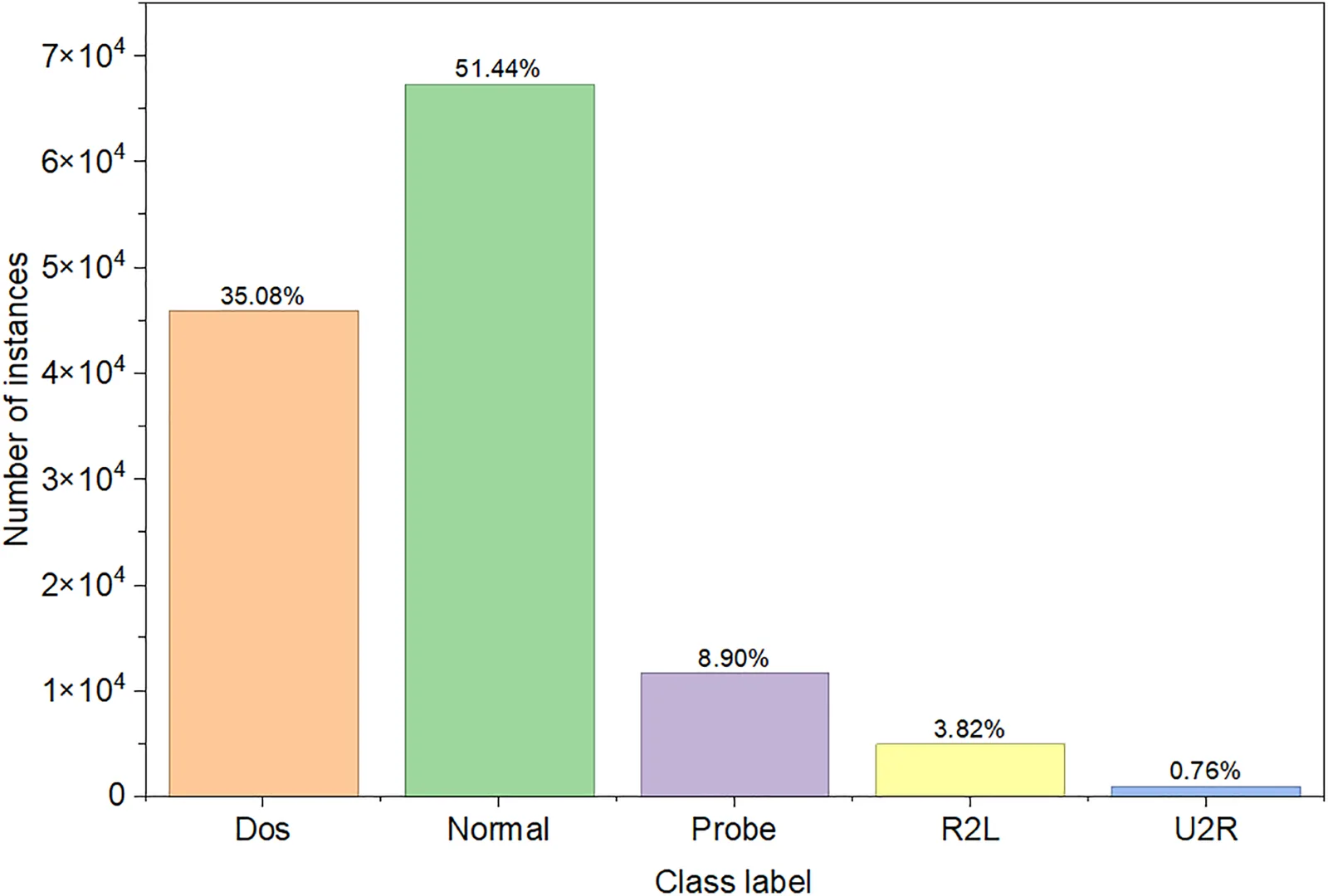
NSL-KDD dataset statistics.

All raw traffic records are first ordered by collection timestamp and partitioned into an 80% training set and a 20% testing set. This chronological split is performed prior to any resampling and any sliding-window construction; consequently, no synthetic sample can cross the training–testing boundary and no sliding window can span across it, thereby preventing information leakage. The complete data preprocessing and partitioning pipeline is summarized in Algorithm 2.

**Algorithm 2.** Data Preprocessing and Partitioning Pipeline

**Input:** Raw traffic datasetD

**Output:** Preprocessed training set $D_{train}$, testing set$D_{test}$

1. Sort D by collection timestamp in ascending order

2. Split D into $D_{train}$ (80%) and $D_{test}$ (20%) chronologically, before any resampling or window construction

3. for $D_{train}$:

    a. Handle missing values using training statistics

    b. Detect and remove outliers based on training distribution

    c. Fit scaler and normalize features

    d. if CICIDS2017: Apply SMOTE + random undersampling

     (i) Apply SMOTE to minority classes and insert each synthetic sample at its interpolated timestamp $t_{syn}=\lambda t_{i}+(\text{1}-\lambda )t_{j}$, with $t_{i}$, $t_{j}$ the timestamps of the two parent samples and λ∈[0,1] the interpolation coefficient, to preserve chronological order

     (ii) Randomly undersample the majority class, preserving the chronological order of retained records, without shuffling

4. for $D_{test}$:

    a. Apply trained imputer from Step 3a

    b. Apply trained scaler from Step 3c

    c. No SMOTE or undersampling is performed

5. return $D_{train}$ and$D_{test}$

Since both resampling operations and sliding-window construction are applied to the chronologically ordered sequence with temporal order strictly enforced throughout the pipeline, the resulting graph sequence preserves valid temporal semantics: each window contains ω consecutive traffic records, and successive windows faithfully reflect the original progression of traffic states.

#### 5.1.2. Performance assessment metrics.

Model performance was quantitatively assessed using a set of established classification metrics, which are derived from the fundamental counts of false positives (FP), false negatives (FN), true positives (TP), and true negatives (TN). These metrics are defined as follows:

1. Accuracy:$A\text{cc}=\frac{TP+TN}{TP+TN+FP+FN}$
2. Precision:$Pre=\frac{TP}{TP+FP}$
3. Recall:$Re=\frac{TP}{TP+FN}$
4. F1-score:$F\text{1}=\text{2}\times \frac{Pre\times Re}{Pre+Re}$
5. False positive rate:$FPR=\frac{FP}{FP+TN}$

#### 5.1.3. Hyperparameter configuration and selection.

To systematically investigate the influence of core hyperparameters on the model performance, sensitivity analysis was performed. In addition to the above assessment metrics, model inference latency was also considered. Hyperparameter tuning is conducted exclusively on a validation set (20% hold-out from the training partition), and the test set is used only for final evaluation.

The number of nearest-neighbor connections per node is a core parameter governing the construction of the dynamic graph topology, as it directly determines the density of the graph structure and the capacity to model spatial topological features. An inappropriate value may lead to either incomplete characterization of topological associations among nodes or feature over-smoothing during graph convolution. As shown in Tables 1 and 2, the core detection metrics on both benchmark datasets exhibit a clear upward trend as the connection number increases from 5 to 10. At a connection number of 10, the model attains peak performance on both datasets, while the per-sample inference time (PIT) remains controlled at 0.09 ms, satisfying the real-time requirements for intrusion detection on power grid edge devices. When the connection number exceeds 10, detection performance on both datasets declines progressively, accompanied by a marked increase in inference time. Notably, the CICIDS2017 dataset contains more weakly correlated associations in the high-dimensional feature space, resulting in a more noticeable performance degradation. Balancing detection performance and inference cost, the optimal count of nearest-neighbor connections is ultimately determined to be 10.

**Table 1:** Model performance at different top-*k* neighbors on the NSL-KDD dataset.

κ	Pre (%)	Re (%)	Acc (%)	F1 (%)	PIT (ms)
5	99.33 ± 0.04	99.32 ± 0.05	99.35 ± 0.04	99.32 ± 0.05	0.070 ± 0.002
10	100.00 ± 0.00	100.00 ± 0.00	100.00 ± 0.00	100.00 ± 0.00	0.090 ± 0.002
15	99.96 ± 0.02	99.95 ± 0.02	99.96 ± 0.01	99.95 ± 0.02	0.100 ± 0.002
20	99.90 ± 0.02	99.89 ± 0.02	99.91 ± 0.02	99.89 ± 0.02	0.120 ± 0.002
25	99.83 ± 0.02	99.82 ± 0.02	99.84 ± 0.02	99.82 ± 0.02	0.150 ± 0.002

Values are reported as mean ± standard deviation over five independent runs.

**Table 2:** Model performance at different top-*k* neighbors on the CICIDS2017 dataset.

κ	Pre (%)	Re (%)	Acc (%)	F1 (%)	PIT (ms)
5	99.25 ± 0.03	99.22 ± 0.03	99.27 ± 0.03	99.24 ± 0.02	0.080 ± 0.002
10	99.97 ± 0.02	99.97 ± 0.02	99.98 ± 0.01	99.98 ± 0.01	0.090 ± 0.002
15	99.91 ± 0.02	99.90 ± 0.02	99.92 ± 0.02	99.90 ± 0.02	0.110 ± 0.002
20	99.84 ± 0.02	99.82 ± 0.02	99.86 ± 0.02	99.83 ± 0.02	0.120 ± 0.002
25	99.77 ± 0.02	99.74 ± 0.02	99.79 ± 0.02	99.75 ± 0.02	0.150 ± 0.002

Values are reported as mean ± standard deviation over five independent runs.

Similarly, the optimal sliding window size is set to *ω* = 80 with a stride of *δ* = 40, yielding a 50% overlap between consecutive windows. The optimal initial learning rate is determined to 0.0001, and the optimal encoder depth is established as 4. These parameters, along with other key hyperparameters systematically tuned to ensure optimal convergence and performance, are listed in Table 3.

**Table 3:** Model hyperparameter configuration.

Hyperparameter	Value
Threshold (θ)	0.5
Epochs (*E*)	20
Learning rate (*L*_*r*_)	0.0001
Optimizer	Adam
Transformer encoder layers (${ℒ}_{u}$)	4
Similarity percentile (π)	0.05
Top-*k* neighbors (κ)	10
Batch size (ℬ)	32
Attention heads	8
GCN hidden dim (*d*_*g*_)	32
Transformer hidden dim (*d*_*u*_)	32
FCN hidden dim	128

#### 5.1.4. Training convergence analysis.

The convergence behavior during model training is a key indicator for assessing learning stability and generalization capability. Figs 8 and 9 present the training and testing loss and accuracy curves of GT-PNID on the NSL-KDD and CICIDS2017 datasets, respectively. Overall, the model converges rapidly during training: the loss function declines sharply in the initial phase and stabilizes thereafter, while the testing loss does not increase with additional training epochs, indicating the absence of overfitting. Moreover, the accuracy on both the training and testing sets reaches a high level early in training and remains consistent throughout subsequent iterations, further validating the model’s favorable learning stability and generalization capability across diverse attack scenarios.

**Fig 8:**
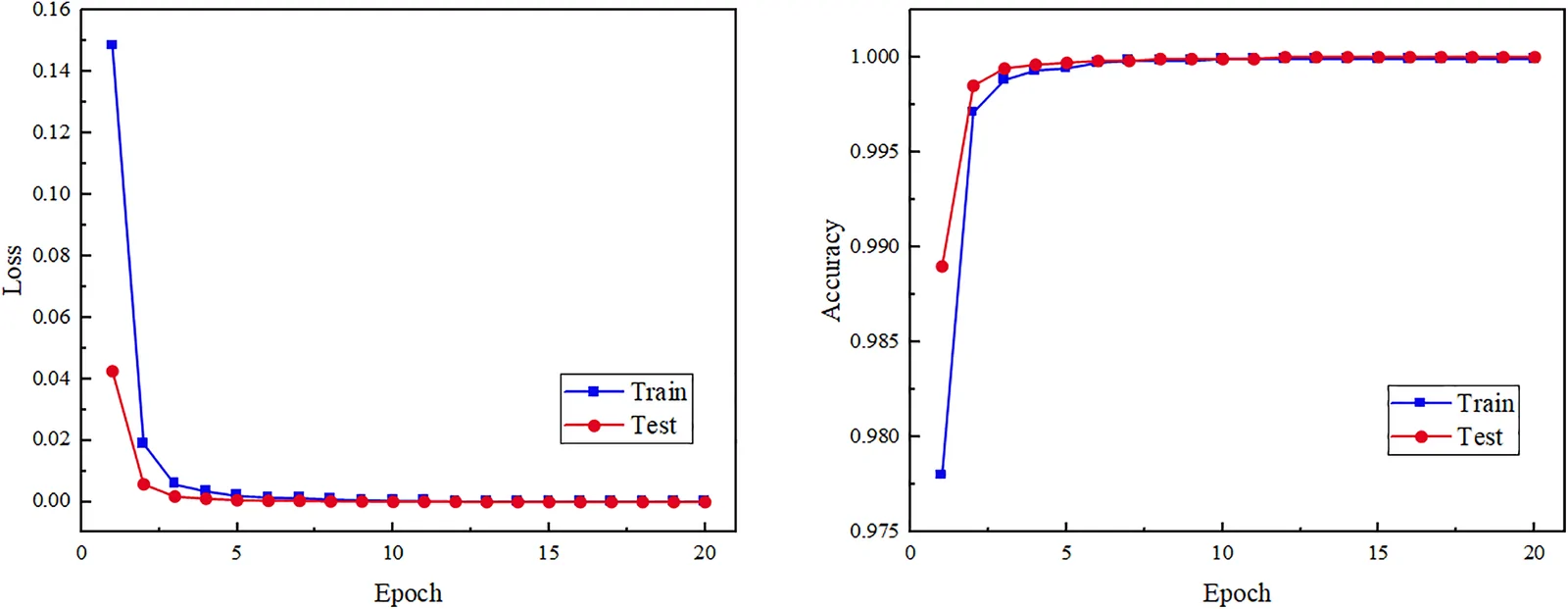
Training and testing performance curves of GT-PNID on NSL-KDD.

**Fig 9:**
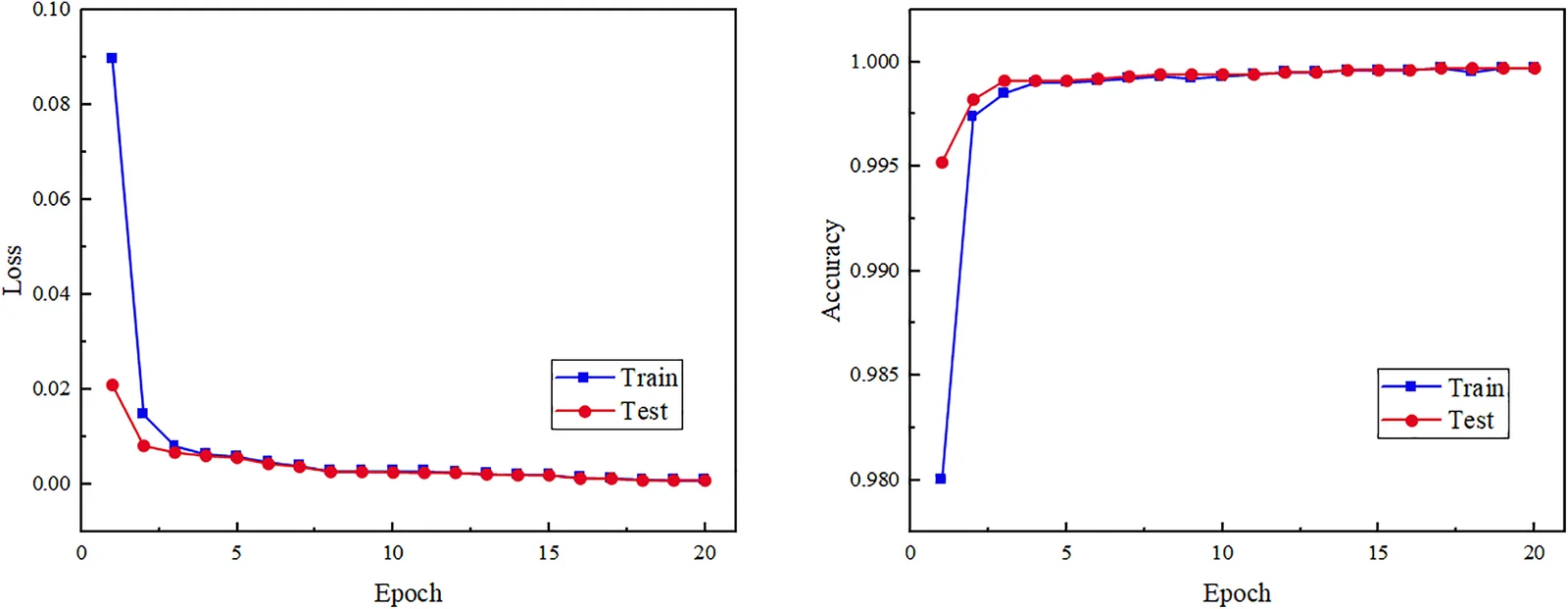
Training and testing performance curves of GT-PNID on CICIDS2017.

### 5.2. Ablation study

To substantiate the individual contribution of each architectural module, an ablation study was conducted on both benchmark datasets, comparing five variants as illustrated in Fig 10: (i) GCN-only: the Transformer temporal encoder and feature fusion module are removed, leaving only the two-layer GCN for spatial encoding followed directly by classification; (ii) GAT-only: identical to (i) but replacing the GCN with a two-layer graph attention network (GAT); (iii) Transformer-only: the GCN encoder is removed, and the node features (without graph convolution) are fed directly into the Transformer temporal encoder; (iv) GAT + TF: the proposed Transformer encoder and feature fusion mechanism are retained, but the GCN is replaced by GAT; and (v) GT-PNID (GCN + TF): the full proposed architecture incorporating GCN and Transformer.

**Fig 10:**
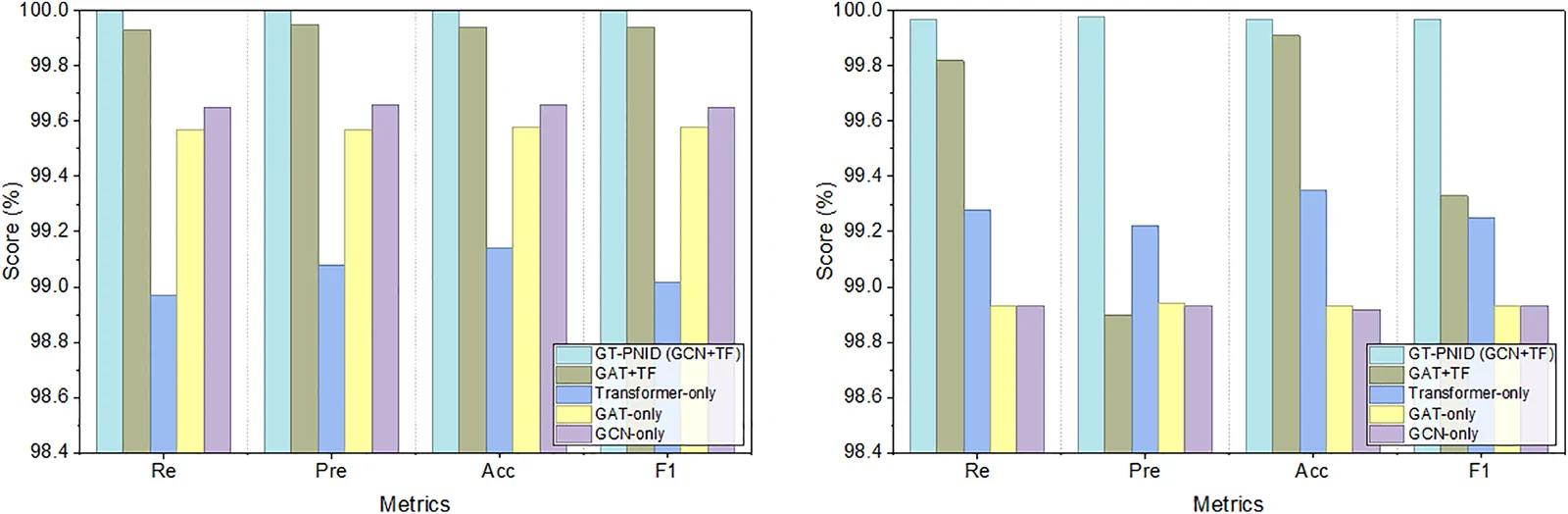
Ablation study results comparing five architectural variants on the (*left*) NSL-KDD and (*right*) CICIDS2017 datasets.

All five variants share the same dynamic graph construction module as the input representation, ensuring that performance differences stem solely from the architectural components under comparison.

As depicted in Fig 10, all spatiotemporal integration variants (GT-PNID and GAT + TF) significantly outperform both the GNN-only variants (GCN-only and GAT-only) and the Transformer-only variant across both datasets. This confirms the superiority of spatiotemporal integration: by combining local graph-based feature extraction with the Transformer’s long-range temporal contextual modeling, a synergistic effect is realized that neither branch alone can achieve. Although the performance of GAT + TF is closely comparable to that of GT-PNID on the relatively simpler NSL-KDD dataset, a discernible performance gap emerges on the more complex CICIDS2017 dataset. This discrepancy can be attributed to the localized neighborhood aggregation in GCNs, which enables more effective learning and generalization over complex graph topologies compared to the adaptive attention mechanism in GAT. The latter, however, may be more susceptible to noise in complex networks. Consequently, GT-PNID achieves the highest performance metrics on both datasets, underscoring the complementary strengths of its dual-component architecture: GCNs for capturing local structural features and Transformers for modeling long-range temporal patterns. The dedicated feature fusion mechanism further enhances this integration, as evidenced by the consistent superiority of GT-PNID over single-branch variants.

Tables 4 and 5 report the per-class performance. On NSL-KDD, GT-PNID achieves near-perfect accuracy and F1-score (99.98%) across all classes, including minority attacks (R2L, U2R), with consistently low false positive rates (FPRs). On the more complex CICIDS2017 dataset, the model maintains superior overall performance, with most categories (Bot, DDoS, DoS variants) achieving outstanding results. Two exceptions warrant particular attention: Heartbleed and Brute Force both exhibit lower recall—the former due to extreme sample scarcity, the latter due to its high behavioral similarity to legitimate authentication traffic, which increases the likelihood of missed detections. Notably, near-zero FPRs across most classes on both datasets are critical for power systems, where false alarms may trigger unnecessary protective actions or operator alarm fatigue.

**Table 4:** Per-class performance of the proposed model on NSL-KDD.

Attack Type	Pre (%)	Re (%)	Acc (%)	F1 (%)	FPR (%)
Normal	99.99	100.00	99.98	99.98	0.01
Dos	100.00	100.00	100.00	100.00	0.00
Probe	99.99	99.99	99.99	99.99	0.01
R2L	100.00	100.00	100.00	100.00	0.00
U2R	100.00	100.00	100.00	100.00	0.00

**Table 5:** Per-class performance of the proposed model on CICIDS2017.

Attack Type	Pre (%)	Re (%)	Acc (%)	F1 (%)	FPR (%)
Benign	99.98	100.00	99.99	99.98	0.02
Bot	100.00	100.00	100.00	100.00	0.00
Brute Force	99.72	97.51	99.95	98.59	0.00
DDos	100.00	99.83	99.98	99.91	0.00
Dos GoldenEye	100.00	100.00	100.00	100.00	0.00
Dos Hulk	99.87	100.00	99.98	99.96	0.03
Dos Slow htt pt	99.78	100.00	99.98	99.91	0.00
Dos Slowloris	100.00	99.74	99.98	99.90	0.00
FTP-patator	100.00	99.76	99.99	99.89	0.00
Heartbleed	100.00	97.58	99.99	98.79	0.00
Infiltration	100.00	100.00	100.00	100.00	0.01
Portcan	100.00	100.00	100.00	100.00	0.01
SSH-patator	99.73	100.00	99.99	99.86	0.01
SQL Injection	100.00	100.00	100.00	100.00	0.00
XSS	99.51	99.76	99.91	99.64	0.00

### 5.3. Comparative evaluation

#### 5.3.1. Comparative evaluation against baseline methods.

To systematically evaluate the superior performance of GT-PNID, multiple mainstream methods covering traditional machine learning and classical deep learning were selected as baselines. Comparative experiments were performed on the NSL-KDD and CICIDS2017 datasets. The baselines include decision tree (DT), random forest (RF), k-nearest neighbors (KNN), SVM, CNN, LSTM, VGG16, and a standalone Transformer model. All baseline models were configured with domain-standard optimal parameters and evaluated under the same data splits, preprocessing pipelines, and experimental conditions to ensure fair and reproducible comparisons. Results are reported in Tables 6 and 7.

**Table 6:** Comparative evaluation against baseline methods on NSL-KDD.

Method	Pre (%)	Re (%)	Acc (%)	F1 (%)	FPR (%)
DT	96.83	97.02	97.15	96.92	3.16
RF	98.04	98.17	98.26	98.1	1.95
SVM	97.28	97.35	97.52	97.31	2.71
KNN	95.72	96.05	96.38	95.88	4.27
CNN	98.65	98.79	98.87	98.72	1.34
LSTM	98.31	98.45	98.52	98.38	1.68
VGG16	98.55	98.63	98.71	98.59	1.45
Transformer	98.97	99.08	99.14	99.02	0.92
GT-PNID	100	100	100	100	0

**Table 7:** Comparative evaluation against baseline methods on CICIDS2017.

Method	Pre (%)	Re (%)	Acc (%)	F1 (%)	FPR (%)
DT	97.63	97.81	98.07	97.72	2.36
RF	98.52	98.46	98.75	98.49	1.53
SVM	98.93	99.46	98.92	99.19	1.07
KNN	96.85	96.52	97.24	96.68	3.47
CNN	99.15	99.08	99.21	99.11	0.89
LSTM	98.96	98.89	99.03	98.92	1.04
VGG16	99.1	99.05	99.17	99.07	0.93
Transformer	99.28	99.22	99.35	99.25	0.71
GT-PNID	99.97	99.97	99.98	99.98	0.03

As shown in Table 6, traditional machine-learning methods are constrained by manual feature engineering, resulting in limited feature representation and classification capacity for network attacks. The accuracy of KNN, DT, and SVM models ranges from 96.38% to 97.52%, with the highest FPR reaching 4.27%, falling short of the low-false-alarm and high-reliability requirements of power-grid scenarios. RF improves stability through ensemble learning, achieving 98.26% accuracy, but still exhibits noticeable false-alarm and missed-detection issues. Among deep-learning baselines, CNN and VGG16 attain accuracies of 98.87% and 98.71%, respectively, by leveraging local feature extraction; LSTM, which specializes in temporal dependency modeling, reaches 98.52%; and the standalone Transformer model, with its global attention mechanism, achieves 99.14% accuracy—the highest among all baselines. However, none of these models jointly models spatial and temporal features, making them unable to fully adapt to the dynamic distribution of attack behaviors in power-grid communication networks. The proposed GT-PNID model achieves perfect scores on this dataset—100.00% for precision, recall, accuracy, and F1-score, with an FPR of 0.00%—comprehensively outperforming all baseline models and enabling error-free identification of various attacks.

As shown in Table 7, on the more diverse and feature-complex CICIDS2017 dataset, the performance trends of each model are similar to those on NSL-KDD. Among traditional machine-learning models, SVM and RF show relatively prominent results with accuracies of 98.92% and 98.75%, respectively, yet still struggle with high-dimensional heterogeneous traffic and class imbalance. Among deep-learning models, CNN and VGG16 achieve 99.21% and 99.17% accuracy, LSTM reaches 99.03%, and the standalone Transformer model, through its long-range dependency modeling, attains 99.35% accuracy, representing the best baseline performance. Nevertheless, single-structure models remain unable to jointly capture spatial-topological associations and global temporal evolution. The proposed GT-PNID model achieves 99.98% accuracy, 99.97% precision and recall, 99.98% F1-score, and an FPR of only 0.03% on this dataset, significantly surpassing all comparison methods and demonstrating high precision and robustness in complex network environments.

#### 5.3.2. Comparative evaluation against state-of-the-art methods.

To validate the efficacy of GT-PNID against leading-edge approaches, a comparative performance evaluation was conducted on CICIDS2017. Table 8 summarizes the results on CICIDS2017. As shown, GT-PNID achieves superior performance, achieving the highest recorded metrics. A key practical advantage lies in its considerably lower computational cost: training requires only 283 seconds, while the average per-sample inference time (PIT) is an exceptionally fast 0.09 milliseconds. This sub-millisecond latency meets the stringent latency budget for in-line traffic inspection in central substation deployment scenarios, where high-throughput and deterministic real-time processing is critical for large-scale network monitoring. Although models such as XGBoost, IoVST, and CNN demonstrate competitive detection rates, they incur substantially greater computational overhead.

**Table 8:** Comparative evaluation against state-of-the-art methods on CICIDS2017.

Method	Pre (%)	Re (%)	Acc (%)	F1 (%)	Training duration (s)	PIT (ms)
CNN [32]	99.82	99.83	99.82	99.84	2668	1.50
HNN [33]	–	–	99.87	99.90	–	–
IoVST [34]	99.82	99.86	99.90	99.82	–	0.71
SVM-B [35]	98.93	99.46	98.92	–	–	–
XGBoost [36]	99.90	99.90	99.90	99.90	1620	0.20
GT-PNID	99.97	99.97	99.98	99.98	283	0.09

### 5.4. Model efficiency assessment

The computational cost of GT-PNID was comprehensively evaluated, encompassing model size, number of parameters, FLOPs, and inference time. This assessment is essential for evaluating its suitability for deployment in real-world, computationally limited operational settings, such as grid edge gateways. The quantitative comparison presented in Table 9 highlights the model’s efficiency.

**Table 9:** Comparison of model efficiency.

Model	Model size (MB)	Total Parameters	FLOPs (M)	PIT (ms)
IoVST [34]	15.1	899,682	–	0.71
STC-GraphFormer [37]	–	1,031,000	1250	0.53
Rec-CNN [38]	–	534,225	500	0.17
GT-PNID	2.21	424,478	9.13	0.09

The on-disk model size of GT-PNID is only 2.21 MB, significantly smaller than 15 MB for IoVST. This compact footprint enables more flexible deployment and updating on resource-constrained edge devices. In terms of parameter count, GT-PNID contains only 424,478 trainable parameters, far fewer than Rec-CNN (534,225), IoVST (899,586), and STC-GraphFormer (1,031,000). The smaller parameter scale not only reduces storage and computational overhead but also allows for faster adaptation and iteration to address emerging attack patterns.

FLOPs serve as a core metric for measuring the computational complexity of a model. As shown in Table 9, the FLOPs of GT-PNID are only 9.13M, which is significantly lower than those of Rec-CNN (500M) and STC-GraphFormer (1250M). This result demonstrates that the model requires substantially less computation during the inference phase, enabling more efficiently operation on embedded platforms.

Inference time serves as a direct indicator for evaluating model real-time performance. Experiments measured the per-sample inference time of GT-PNID to be 0.09 ms, which is considerably faster than the comparison models. This sub-millisecond latency sufficiently meets the millisecond-level response requirements of monitoring scenarios in power systems.

Overall, GT-PNID demonstrates significant advantages in computational efficiency, model lightweighting, and real-time performance. These results indicate that it can provide efficient and reliable intrusion detection on hardware-constrained substation edge devices, highlighting its strong potential for grid security applications.

### 5.5. Edge platform deployment validation

The primary deployment platform for power grid intrusion detection typically consists of resource-constrained edge computing nodes that impose stringent real-time processing requirements. As demonstrated in prior analyses, the proposed model exhibits characteristics amenable to lightweight deployment. To comprehensively evaluate its practical performance in a genuinely constrained environment, this work deployed and tested the model on an actual embedded edge platform—an NVIDIA Jetson TX2 system employed for on-site safety monitoring in power grid operations, as shown in Fig 11. This deployment enables an assessment of the model’s inference performance and engineering feasibility under realistic edge constraints.

**Fig 11:**
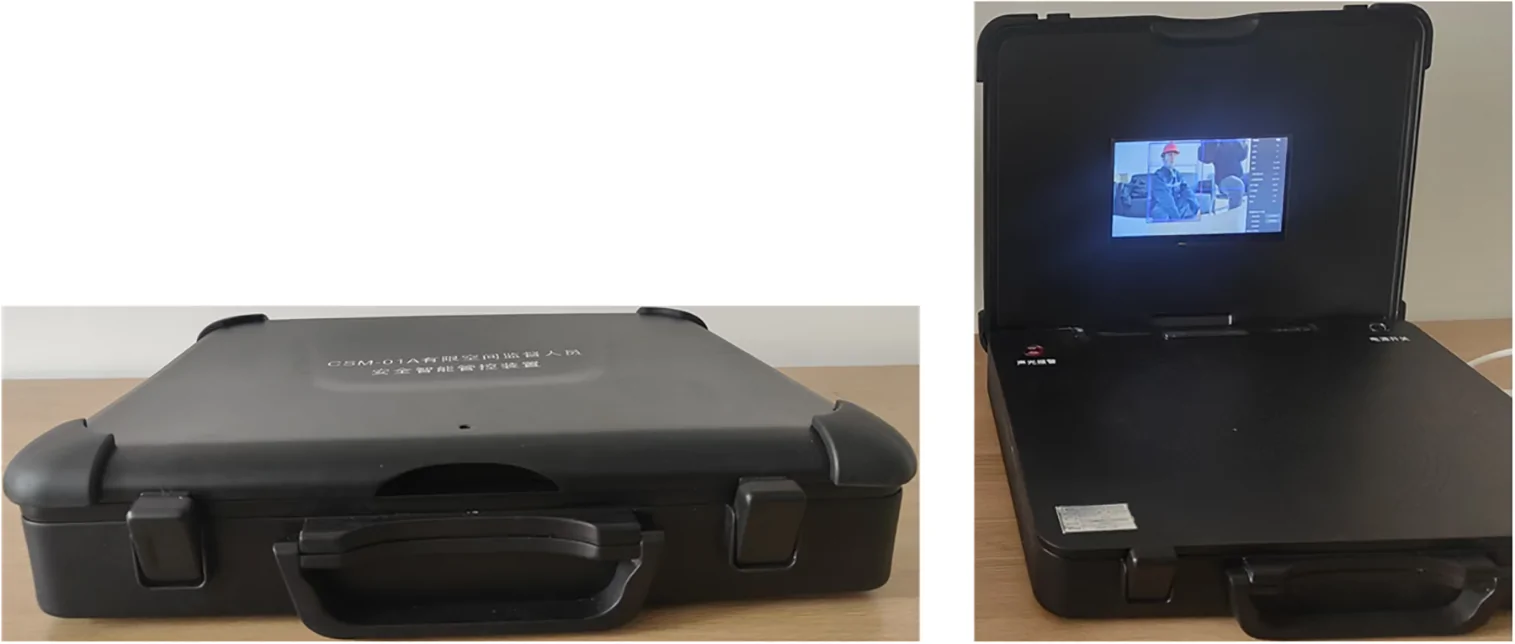
Edge platform used for testing. (Left) physical appearance of the platform, (Right) platform in operation.

The inference performance of the model on the edge platform (Jetson TX2) was benchmarked against a conventional server platform (RTX 4060) using the CICIDS2017 dataset. Each platform performed 1,000 independent inference runs, with the average inference time recorded as the primary performance metric. Comparative results are presented in Table 10.

**Table 10:** Comparative evaluation of GT-PNID on edge and server platforms using the CICIDS2017 dataset.

Hardware Platform	Acceleration Mode	Inference Time (ms)	GPU Memory Usage (MB)	Accuracy (%)
RTX 4060	PyTorch (FP32)	0.09	120	99.98
Jetson TX2	PyTorch (FP32)	3.21	168	99.98
Jetson TX2	TensorRT (FP16)	1.68	110	99.97

The experimental results in Table 10 demonstrate the proposed model’s strong practical adaptability in edge-computing environments. Even under PyTorch’s default FP32 inference mode on the Jetson TX2 platform, the model achieves a per-sample inference time of only 3.21 ms; when accelerated with TensorRT in FP16 mode, inference time drops further to 1.68 ms, nearly doubling throughput and confirming high real-time capability. The GPU memory footprint remains low (110–170 MB), far below the platform’s 8 GB total capacity, indicating efficient utilization of limited edge resources. Moreover, FP32 accuracy on the edge platform matches that on the server (99.98%); even with FP16 half-precision inference, accuracy degrades only by 0.01%, reflecting robust tolerance to quantization and validating the feasibility of accelerated half-precision inference without substantial performance loss in deployment.

We note that while the 1.68 ms latency achieved under TensorRT FP16 fully enables real-time edge monitoring, it does not yet meet the strict sub-millisecond latency budget of protection-grade functions; bridging this gap through further model acceleration techniques—such as INT8 quantization, structured pruning, and knowledge distillation—is left as future work.

## 6. Conclusions

This paper presents GT-PNID, a graph-temporal network designed to address the distinctive intrusion detection challenges of power communication networks, including protocol heterogeneity, stringent real-time constraints, and zero-tolerance for false positives. The model employs a dynamic graph construction mechanism to transform sequential network traffic flows into spatiotemporal graph representations, which are jointly processed by a GCN encoder for local spatial correlations and a stacked Transformer encoder for long-range temporal dependencies, with a dedicated aggregation mechanism integrating both outputs for synergistic spatiotemporal analysis.

Comprehensive empirical evaluations conducted on two distinct public benchmark datasets, CICIDS2017 and NSL-KDD, demonstrate that GT-PNID achieves average accuracy and F1-scores exceeding 99.98% on both datasets, outperforming several established and state-of-the-art alternative approaches. These findings validate its efficacy in jointly learning spatial and temporal features and underscore its strong generalization potential across diverse network environments. Notably, the model exhibits operational efficiency, comprising only approximately 424 thousand trainable parameters and achieving an inference time of roughly 0.09 milliseconds per sample, thereby confirming its suitability for real-time IDS within the computationally limited settings typical of operational power grid communication infrastructures. Collectively, these attributes position GT-PNID as a robust foundational framework for practical power network IDS.

Future research will proceed along four directions.

1) The model will be validated and fine-tuned on real or high-fidelity simulated power network traffic containing IEC 61850 GOOSE/SV, MMS, DNP3, and IEC 60870-5-104 protocol flows, with labeled attacks such as protocol-level message spoofing and injection.
2) Class imbalance handling warrants further investigation: sample-level SMOTE merely approximates the temporal structure of the training sequence, and future work will explore two alternative strategies—window-level oversampling, which retains intra-window temporal order and class-weighted/focal loss, which preserves the training data in its original form.
3) Future work will investigate advanced model compression and lightweight acceleration techniques—including INT8 quantization, structured pruning, and knowledge distillation—to further reduce computational overhead and enable deployment on resource-constrained edge devices, as well as semi-supervised and unsupervised learning paradigms to improve detection capability for novel, unseen attack types.
4) The graph construction module will be extended by incorporating physical grid topology constraints, including substation connectivity and power flow equations, thereby evolving GT-PNID into a cyber-physical IDS capable of detecting coordinated attacks that violate underlying physical laws, such as state estimation-oriented false data injection attacks.

### Notations

Notations used in this paper are listed below:

**Table:** 

Symbol	Interpretation
${\text{A}}^{\left(i\right)}$	Spatiotemporal feature data for the *i*-th sample
$c^{\left(i\right)}$	Label vector for the i-th sample
${𝐀}_{u}$	Node feature matrix for the u-th time window
${𝐜}_{u}$	Label set for the u-th time window
𝔊	Sequence of dynamic graph structures
$G_{u}$	Graph snapshot at the u-th time window
$V_{u}$	Vertex set of graph$G_{u}$
$E_{u}$	Edge set of graph$G_{u}$
$\Gamma_{u}$	Edge weight attribute matrix of graph$G_{u}$
$c_{u}$	Graph-level label for windowu
$H_{g}$	Output of the graph convolutional encoder
$H_{u}$	Output of the temporal sequence encoder
$L_{u}$	Number of layers in the Transformer encoder

## Supporting information

S1 File Source Code of GT-PNID. (PDF)

S2 File Supplementary sensitivity analysis. (PDF)

S3 File Window purity analysis. (PDF)

S1 Table Time complexity breakdown of GT-PNID. (PDF)

S2 Table Comprehensive deployment runtime log of GT-PNID on Jetson TX2 edge platform. (PDF)

## References

[1] Pollard M. A case study of Russian cyber-attacks on the Ukrainian power grid: Implications and best practices for the United States. Pepperdine Policy Rev. 2024;16(1):1.

[2] Agarwal L, Jaint B, Mandpura AK. Hybrid AI framework for detecting cyberattacks and predicting cascading failures in power systems. Sustain Comput: Inform Syst. 2025;48:101222. doi: 10.1016/j.suscom.2025.101222

[3] Qin Y, Luo Z, Che Y, Cao L. False data injection attacks against UAV Battery state of health estimation with a capacity-informed latent diffusion model. Green Energy Intell Transp. 2026;:100404. doi: 10.1016/j.geits.2026.100404

[4] Sweeten J, Takiddin A, Ismail M, Refaat SS, Atat R. Cyber-Physical GNN-Based Intrusion Detection in Smart Power Grids. In: 2023 IEEE International Conference on Communications, Control, and Computing Technologies for Smart Grids (SmartGridComm). 2023. pp. 1–6.

[5] Gunduz MZ, Das R. Cyber-security on smart grid: Threats and potential solutions. Comput Netw. 2020;169:107094. doi: 10.1016/j.comnet.2019.107094

[6] Fernandes T, Magalhães JP, Alves W. Cybersecurity in smart railways: Exploring risks, vulnerabilities and mitigation in the data communication services. Green Energy Intell Transp. 2025;4(4):100305. doi: 10.1016/j.geits.2025.100305

[7] Ding J, Qammar A, Zhang Z, Karim A, Ning H. Cyber threats to smart grids: Review, taxonomy, potential solutions, and future directions. Energies. 2022;15(18). doi: 10.3390/en15186799

[8] Alani MM, Mauri L, Damiani E. A two-stage cyber attack detection and classification system for smart grids. Internet Things. 2023;24:100926. doi: 10.1016/j.iot.2023.100926

[9] Stryczek S, Natkaniec M. Internet Threat detection in smart grids based on network traffic analysis using LSTM, IF, and SVM. Energies. 2022;16(1):329. doi: 10.3390/en16010329

[10] Havlena V, Matoušek P, Ryšavý O, Holík L. Accurate automata-based detection of cyber threats in smart grid communication. IEEE Trans Smart Grid. 2023;14(3):2352–66. doi: 10.1109/tsg.2022.3216726

[11] Diaba SY, Elmusrati M. Proposed algorithm for smart grid DDoS detection based on deep learning. Neural Netw. 2023;159:175–84. doi: 10.1016/j.neunet.2022.12.011 36577364

[12] Kasongo SM. A deep learning technique for intrusion detection system using a recurrent neural networks based framework. Comput Commun. 2023;199:113–25. doi: 10.1016/j.comcom.2022.12.010

[13] Yuan Y, Guo Y, Dehghanpour K, Wang Z, Wang Y. Learning-based real-time event identification using rich real PMU data. IEEE Trans Power Syst. 2021;36(6):5044–55. doi: 10.1109/tpwrs.2021.3081608

[14] L. Amutha A, Annie Uthra R, Preetha Roselyn J, Golda Brunet R. Anomaly detection and classification in streaming PMU data in smart grids. Comput Syst Sci Eng. 2023;46(3):3387–401. doi: 10.32604/csse.2023.029904

[15] Zhang H, Cheng J, Bai X, Wang D, Gao R, Fan B. A hierarchical spatio-temporal graph attention network for false data injection attack detection in smart grids. Processes. 2026;14(3):507.

[16] M L, Adnan MM, Reddy RA, Vasukidevi G, G A. A Graph Neural Network and Transformer Encoder Technique for Anomaly and Cyber Threat Detection in Smart Grids. In: 2024 International Conference on Intelligent Algorithms for Computational Intelligence Systems (IACIS). 2024. pp. 1–4.

[17] Yuan J, Li Y. An APT attack detection method of a new-type power system based on STSA-transformer. Electr Electron Eng. 2024;17(1):19–28. doi: 10.2174/2352096516666230428104141

[18] Du D, Zhu M, Li X, Fei M, Bu S, Wu L, et al. A review on cybersecurity analysis, attack detection, and attack defense methods in cyber-physical power systems. J Modern Power Syst Clean Energy. 2023;11(3):727–43. doi: 10.35833/mpce.2021.000604

[19] Yin T, Naqvi SAR, Nandanoori SP, Kundu S. Advancing cyber-attack detection in power systems: A comparative study of machine learning and graph neural network approaches. 2024 Resilience Week (RWS). IEEE; 2024.

[20] Alsirhani A, Tariq N, Humayun M, Naif Alwakid G, Sanaullah H. Intrusion detection in smart grids using artificial intelligence-based ensemble modelling. Cluster Comput. 2025;28(4). doi: 10.1007/s10586-024-04964-9

[21] Idowu A, Ismaila I, Adebayo Ojeniyi J. Machine learning-driven cybersecurity solutions for enhanced smart grids and critical infrastructure: A review. NIPES - J Sci Technol Res. 2025;7(3):159–84. doi: 10.37933/nipes/7.3.2025.11

[22] Jithish J, Alangot B, Mahalingam N, Yeo KS. Distributed anomaly detection in smart grids: A federated learning-based approach. IEEE Access. 2023;11:7157–79. doi: 10.1109/access.2023.3237554

[23] Mazhar T, Irfan HM, Khan S, Haq I, Ullah I, Iqbal M, et al. Analysis of cyber security attacks and its solutions for the smart grid using machine learning and blockchain methods. Fut Internet. 2023;15(2):83. doi: 10.3390/fi15020083

[24] Alsaiari A, Ilyas M. A hybrid CNN-LSTM deep learning model for intrusion detection in smart grid. Int J Artif Intell Appl. 2025;16(5):01–22. doi: 10.5121/ijaia.2025.16501

[25] Abdelkhalek M, Ravikumar G, Govindarasu M. Ml-based anomaly detection system for der communication in smart grid. 2022 IEEE power & energy society innovative smart grid technologies conference (ISGT); IEEE; 2022.

[26] Berghout T, Benbouzid M, Amirat Y. Towards resilient and secure smart grids against PMU adversarial attacks: A deep learning-based robust data engineering approach. Electronics. 2023;12(12). doi: 10.3390/electronics12122554

[27] Foggo B, Yamashita K, Yu N. pmuBAGE: The benchmarking assortment of generated PMU data for power system events. IEEE Trans Power Syst. 2024;39(2):3485–96. doi: 10.1109/tpwrs.2023.3280430

[28] Anitha T, Aanjankumar S, Poonkuntran S, Nayyar A. A novel methodology for malicious traffic detection in smart devices using BI-LSTM–CNN-dependent deep learning methodology. Neural Comput Appl. 2023;35(27):20319–38. doi: 10.1007/s00521-023-08818-0

[29] Ullah S, Ahmad J, Khan MA, Alshehri MS, Boulila W, Koubaa A, et al. TNN-IDS: Transformer neural network-based intrusion detection system for MQTT-enabled IoT Networks. Comput Netw. 2023;237:110072. doi: 10.1016/j.comnet.2023.110072

[30] Jafari S, Saber AM, Kundur D. Cyber intrusion detection in protective relays using deep learning attention-based models. In: 2025 IEEE International Conference on Communications, Control, and Computing Technologies for Smart Grids (SmartGridComm). 2025. pp. 1–6.

[31] Vaswani A, Shazeer N, Parmar N, Uszkoreit J, Jones L, Gomez AN. Attention is All You Need. In: Guyon I, Luxburg UV, Bengio S, Wallach H, Fergus R, Vishwanathan S, editors. Advances in Neural Information Processing Systems. Curran Associates, Inc.; 2017.

[32] Yang L, Shami A. A Transfer Learning and Optimized CNN Based Intrusion Detection System for Internet of Vehicles. ICC 2022 - IEEE International Conference on Communications. 2022. pp. 2774–9.

[33] Lei S, Xia C, Li Z, Li X, Wang T. HNN: A novel model to study the intrusion detection based on multi-feature correlation and temporal-spatial analysis. IEEE Trans Netw Sci Eng. 2021;8(4):3257–74. doi: 10.1109/tnse.2021.3109644

[34] Cao J, Di X, Li J, Yu K, Zhao L. IoVST: An anomaly detection method for IoV based on spatiotemporal feature fusion. Fut Gener Comput Syst. 2025;166:107636. doi: 10.1016/j.future.2024.107636

[35] Gu J, Lu S. An effective intrusion detection approach using SVM with naïve Bayes feature embedding. Comput Secur. 2021;103:102158. doi: 10.1016/j.cose.2020.102158

[36] Mokbal F, Dan W, Osman M, Ping Y, Alsamhi S. An efficient intrusion detection framework based on embedding feature selection and ensemble learning technique. Int Arab J Inform Technol. 2022;19(2). doi: 10.34028/iajit/19/2/11

[37] Al-Absi GA, Fang Y, Qaseem AA. STC-GraphFormer: Graph spatial-temporal correlation transformer for in-vehicle network intrusion detection system. Veh Commun. 2025;51:100865. doi: 10.1016/j.vehcom.2024.100865

[38] Desta AK, Ohira S, Arai I, Fujikawa K. Rec-CNN: In-vehicle networks intrusion detection using convolutional neural networks trained on recurrence plots. Veh Commun. 2022;35:100470. doi: 10.1016/j.vehcom.2022.100470

