# Supplementary material for: GT-PNID: A lightweight graph-temporal network integrating GCN and transformer for power network intrusion detection
Source: PLoS One. 2026 Nov 6;21(11):e0360854. doi: 10.1371/journal.pone.0360854 (PMC13742970; doi:10.1371/journal.pone.0360854)
Supplement: Supplementary file 1 [file pone.0360854.s001.pdf]

Table of Contents

1. Environment Configuration 1
2. Data Preprocessing 2
3. Training and Testing Procedure 3
4. Source Code 3

1. Environment Configuration

All training and testing were conducted within Jupyter Notebook. The kernel used is T-GGN, and its corresponding environment was configured via Anaconda Prompt and imported into Jupyter. The detailed procedure is as follows:

(1) Open Anaconda Prompt, activate the T-GGN environment, and install the required core libraries—including pandas, scikit-learn, TensorFlow/PyTorch, NumPy, and Matplotlib—using pip install or conda install commands.

```
torch-scatter             2.1.2+pt24cpu            pypi_0    pypi
torch-sparse              0.6.18+pt24cpu           pypi_0    pypi
torch-spline-conv         1.2.2+pt24cpu            pypi_0    pypi
torchaudio                2.4.0                  py38_cpu    pytorch
torchvision               0.19.0                 py38_cpu    pytorch
tornado                   6.4.1            py38h827c3e9_0    anaconda
tqdm                      4.66.5                   pypi_0    pypi
traitlets                 5.14.3           py38haa95532_0    anaconda
transformers              4.46.2                   pypi_0    pypi
typing_extensions         4.11.0           py38haa95532_0    anaconda
ucrt                      10.0.22621.0         h57928b3_0    conda-forge
urllib3                   2.2.2              pyhd8ed1ab_1    conda-forge
vc                        14.3                h8a93ad2_20    conda-forge
vc14_runtime              14.44.35208         h818238b_31    conda-forge
vcomp14                   14.44.35208         h818238b_31    conda-forge
vs2015_runtime            14.44.35208         h38c0c73_31    conda-forge
wcwidth                   0.2.5              pyhd3eb1b0_0    anaconda
wheel                     0.44.0             pyhd8ed1ab_0    conda-forge
win_inet_pton             1.1.0              pyhd8ed1ab_6    conda-forge
xorg-libxau               1.0.11               hcd874cb_0    conda-forge
xorg-libxdmcp             1.1.3                hcd874cb_0    conda-forge
xz                        5.2.6                h8d14728_0    conda-forge
yaml                      0.2.5                h8ffe710_2    conda-forge
yarl                      1.9.4                    pypi_0    pypi
zeromq                    4.3.5                hd77b12b_0    anaconda
zipp                      3.17.0           py38haa95532_0    anaconda
zstandard                 0.23.0           py38hf92978b_0    conda-forge
zstd                      1.5.6                h0ea2cb4_0    conda-forge

(T-GNN) C:\Users\msi>pip install pandas
```

(2) In Anaconda Prompt, execute the command python -m ipykernel install --user --name T-GNN --display-name "T-GNN" to add the environment to Jupyter's kernel list. Subsequently, select the corresponding kernel in Jupyter.

```
torch-scatter             2.1.2+pt24cpu            pypi_0    pypi
torch-sparse              0.6.18+pt24cpu           pypi_0    pypi
torch-spline-conv         1.2.2+pt24cpu            pypi_0    pypi
torchaudio                2.4.0                  py38_cpu    pytorch
torchvision               0.19.0                 py38_cpu    pytorch
tornado                   6.4.1           py38h827c3e9_0    anaconda
tqdm                      4.66.5                   pypi_0    pypi
traitlets                 5.14.3          py38haa95532_0    anaconda
transformers              4.46.2                   pypi_0    pypi
typing_extensions         4.11.0          py38haa95532_0    anaconda
ucrt                      10.0.22621.0          h57928b3_0    conda-forge
urllib3                   2.2.2               pyhd8ed1ab_1    conda-forge
vc                        14.3                 h8a93ad2_20    conda-forge
vc14_runtime              14.44.35208          h818238b_31    conda-forge
vcomp14                   14.44.35208          h818238b_31    conda-forge
vs2015_runtime            14.44.35208          h38c0c73_31    conda-forge
wcwidth                   0.2.5               pyhd3eb1b0_0    anaconda
wheel                     0.44.0              pyhd8ed1ab_0    conda-forge
win_inet_pton             1.1.0               pyhd8ed1ab_6    conda-forge
xorg-libxau               1.0.11                hcd874cb_0    conda-forge
xorg-libxdmcp             1.1.3                 hcd874cb_0    conda-forge
xz                        5.2.6                 h8d14728_0    conda-forge
yaml                      0.2.5                 h8ffe710_2    conda-forge
yarl                      1.9.4                    pypi_0    pypi
zeromq                    4.3.5                 hd77b12b_0    anaconda
zipp                      3.17.0          py38haa95532_0    anaconda
zstandard                 0.23.0          py38hf92978b_0    conda-forge
zstd                      1.5.6                 h0ea2cb4_0    conda-forge

(T-GNN) C:\Users\msi>python -m ipykernel install --user --name T-GNN --display-name "T-GNN"
```

(3) The core environment and libraries required for this experiment are shown in the figure below:

```python
import os
import gc
import time
import torch
import psutil
import numpy as np
import pandas as pd
import torch.nn as nn
import seaborn as sns
import torch.optim as optim
import torch.nn.functional as F
import matplotlib.pyplot as plt
from collections import Counter
from torch_geometric.data import Data, DataLoader
from imblearn.over_sampling import SMOTE
from sklearn.preprocessing import LabelEncoder, StandardScaler
from imblearn.under_sampling import RandomUnderSampler
from sklearn.model_selection import train_test_split, StratifiedKFold
from sklearn.metrics import (accuracy_score, precision_score, recall_score,
                             f1_score, confusion_matrix, classification_report)
```

2. Data Preprocessing

(1) Copy the storage path of the original dataset in the local file system (e.g., E:/Datas/CICIDS2017) to the data preprocessing code module and execute it.
(2) The system performs data cleaning, data balancing, and data normalization according to the preprocessing code.
(3) The system automatically saves the preprocessed dataset to the specified path.
(4) Visual verification of the preprocessed dataset is conducted; a traffic segment is shown in the figure below.

	Destination Port	Flow Duration	Total Fwd Packets	Total Backward Packets	Total Length of Fwd Packets	Total Length of Bwd Packets	Fwd Packet Length Max	Fwd Packet Length Min	Fwd Packet Length Mean	Fwd Packet Length Std	...	min_seg_size_forward	Active Mean
**0**	-0.451191	-0.454332	-0.011418	-0.009727	-0.052077	-0.007310	-0.237920	0.413150	-0.086115	-0.253430	...	0.002629	-0.129919
**1**	-0.430357	2.881507	0.044123	0.051436	0.029320	0.023394	0.371006	-0.297946	-0.210757	0.031046	...	0.002640	-0.052819
**2**	-0.308774	-0.456401	-0.011418	-0.008756	-0.055759	-0.007368	-0.289340	-0.200979	-0.284216	-0.253430	...	0.002629	-0.129919
**3**	-0.451191	-0.456390	-0.011418	-0.009727	-0.050236	-0.007321	-0.212210	0.720214	0.012936	-0.253430	...	0.002629	-0.129919
**4**	-0.430357	2.942851	0.013123	0.008719	0.210139	-0.004278	0.492791	-0.297946	0.401319	0.524386	...	0.002640	0.246017
**...**	...	...	...	...	...	...	...	...	...	...	...	...	...
**1981267**	-0.449748	-0.293938	-0.008835	-0.009727	-0.056341	-0.007373	-0.297459	-0.297946	-0.315495	-0.253430	...	0.002640	-0.129919
**1981268**	-0.449748	-0.309340	-0.008835	-0.009727	-0.056341	-0.007373	-0.297459	-0.297946	-0.315495	-0.253430	...	0.002640	-0.129919
**1981269**	-0.449748	-0.298001	-0.008835	-0.009727	-0.056341	-0.007373	-0.297459	-0.297946	-0.315495	-0.253430	...	0.002640	-0.129919
**1981270**	-0.449748	-0.294389	-0.008835	-0.009727	-0.056341	-0.007373	-0.297459	-0.297946	-0.315495	-0.253430	...	0.002640	-0.129919
**1981271**	-0.449748	-0.300582	-0.008835	-0.009727	-0.056341	-0.007373	-0.297459	-0.297946	-0.315495	-0.253430	...	0.002640	-0.129919

3. Training and Testing Procedure

(1) Launch the system training and testing module, and select the corresponding dataset (e.g., CICIDS2017).
(2) Load the model and initialize its parameters.
(3) The model completes training and testing on the specified dataset according to the training script.
(4) Upon completion of training, execute the code to save the model parameters as a local file.
(5) The model outputs performance evaluation metrics, including accuracy and false positive rate.

4. Source Code

```
import os
import gc
import time
import torch
import psutil
import numpy as np
import pandas as pd
import torch.nn as nn
import seaborn as sns
import torch.optim as optim
import torch.nn.functional as F
import matplotlib.pyplot as plt
from collections import Counter
from torch_geometric.data import Data, DataLoader
from imblearn.over_sampling import SMOTE
from sklearn.preprocessing import LabelEncoder, StandardScaler
from imblearn.under_sampling import RandomUnderSampler
from sklearn.model_selection import train_test_split, StratifiedKFold
```

```
from sklearn.metrics import (accuracy_score, precision_score, recall_score,
                             f1_score, confusion_matrix, classification_report)


# ------------------------- Configuration parameter definition -------------------------
class Config:
    """ Model and training parameter configuration class """
    def __init__(self):
        # Data processing parameters
        self.window_size = 80
        self.step_size = 40
        self.test_ratio = 0.2
        self.random_state = 42

        # Model parameters
        self.gcn_hidden_dim1 = 64
        self.gcn_hidden_dim2 = 32
        self.transformer_heads = 16
        self.transformer_layers = 4
        self.transformer_ffn_dim = 512
        self.dropout_rate = 0.5

        # Train parameters
        self.batch_size = 32
        self.epochs = 20
        self.learning_rate = 0.0001
        self.weight_decay = 1e-5

        # Path parameters
        self.cicids_raw_path = 'E:/Datas/total.TGmodel/new.total.csv'
        self.cicids_processed_path = 'E:/new_data_2017/new_processed_data.csv'

    def print_config(self):
        """ Print configuration parameters """
        print("\n===== Configuration parameters =====")
        for key, value in self.__dict__.items():
            print(f"{key}: {value}")
        print("===================")


# ------------------------- General function for data processing -------------------------
def load_data(file_path):
    try:
        data = pd.read_csv(file_path)
```

```
        print(f"Data loaded successfully, shape: {data.shape}")
        return data
    except Exception as e:
        print(f"Error loading data: {str(e)}")
        raise


def check_data_quality(data):
    # Check class distribution
    print(f"\nClass distribution:\n{data.iloc[:, -1].value_counts()}")

    # Check duplicate rows
    dup_rows = data[data.duplicated()].shape[0]
    print(f"Number of duplicate rows: {dup_rows}")

    # Check NaN and Inf values
    print(f"Total NaN values: {data.isna().sum().sum()}")
    inf_count = (data == np.inf).sum().sum() + (data == -np.inf).sum().sum()
    print(f"Total Inf values: {inf_count}")


def remove_duplicates(data):
    label_col = data.columns[-1]
    benign_data = data[data[label_col] == 'BENIGN'].drop_duplicates()
    other_data = data[data[label_col] != 'BENIGN']
    cleaned_data = pd.concat([benign_data, other_data], ignore_index=True)
    print(f"Duplicates removed. New shape: {cleaned_data.shape}")
    return cleaned_data


def handle_outliers(data):
    data.replace([np.inf, -np.inf], 0, inplace=True)
    data.fillna(0, inplace=True)
    return data


def clean_data(data):
    data = remove_duplicates(data)
    data = handle_outliers(data)
    return data


def remove_zero_variance_features(X):
    variances = X.var()
```

```python
    zero_var_cols = variances[variances == 0].index
    if len(zero_var_cols) > 0:
        X = X.drop(columns=zero_var_cols)
        print(f"Removed {len(zero_var_cols)} zero-variance features. Remaining: {X.shape[1]}")
    return X


def normalize_features(X):
    scaler = StandardScaler()
    X_scaled = scaler.fit_transform(X)
    return pd.DataFrame(X_scaled, columns=X.columns), scaler


def encode_labels(y):
    encoder = LabelEncoder()
    y_encoded = encoder.fit_transform(y)
    return y_encoded, encoder


def process_features(data):
    label_col = data.columns[-1]
    X = data.iloc[:, :-1]
    y = data[label_col]

    X = remove_zero_variance_features(X)
    X, scaler = normalize_features(X)
    y_encoded, encoder = encode_labels(y)

    return pd.concat([X, pd.Series(y_encoded, name=label_col)], axis=1), scaler, encoder


def balance_classes(data, config):
    label_col = data.columns[-1]
    X = data.iloc[:, :-1]
    y = data[label_col]

    # Undersample majority class (BENIGN)
    rus = RandomUnderSampler(
        sampling_strategy={0: Counter(y)[0] // 2},
        random_state=config.random_state
    )
    X_resampled, y_resampled = rus.fit_resample(X, y)
    print(f"After undersampling: {Counter(y_resampled)}")
```

```
    # Oversample minority classes
    target_counts = {
        **{cls: 50000 for cls in [3, 7, 6, 5, 11, 1, 12, 14]},
        **{cls: 5000 for cls in [9, 13, 8]}
    }
    smote = SMOTE(
        sampling_strategy=target_counts,
        random_state=config.random_state
    )
    X_balanced, y_balanced = smote.fit_resample(X_resampled, y_resampled)

    print(f"After oversampling: {Counter(y_balanced)}, Total samples: {X_balanced.shape[0]}")
    return pd.concat([X_balanced, pd.Series(y_balanced, name=label_col)], axis=1)


# -------------------------- Graph data constructor function --------------------------
def calculate_similarity(node_features, window_features):
    return np.linalg.norm(window_features - node_features, axis=1)


def create_edges(window_features, num_neighbors=10, threshold=0.5):
    edge_index = []
    edge_weight = []
    window_size = window_features.shape[0]

    for i in range(window_size):
        node_features = window_features[i]
        similarity_row = calculate_similarity(node_features, window_features)
        sorted_indices = np.argsort(similarity_row)

        for j in sorted_indices[1:num_neighbors + 1]:
            if similarity_row[j] < threshold and j != i:
                edge_index.append((i, j))
                edge_weight.append(1 - similarity_row[j])

    return edge_index, edge_weight


def determine_graph_label(window_labels):
    if np.all(window_labels == 0):
        return 0
    else:
        attack_labels = window_labels[window_labels != 0]
        return np.bincount(attack_labels).argmax() if len(attack_labels) > 0 else 0
```

```
def create_graphs(features, labels, config):
    graphs = []
    window_size = config.window_size
    step_size = config.step_size

    for start in range(0, len(features) - window_size + 1, step_size):
        end = start + window_size
        window_features = features[start:end]
        window_labels = labels[start:end]

        graph_label = determine_graph_label(window_labels)
        edge_index, edge_weight = create_edges(window_features)

        # Convert to PyTorch Geometric format
        edge_index = torch.tensor(edge_index, dtype=torch.long).t().contiguous()
        edge_weight = torch.tensor(edge_weight, dtype=torch.float32)

        graph = Data(
            x=torch.tensor(window_features, dtype=torch.float32),
            edge_index=edge_index,
            edge_attr=edge_weight,
            y=torch.tensor([graph_label], dtype=torch.long)
        )
        graphs.append(graph)

    print(f"Created {len(graphs)} graphs")
    return graphs


# ------------------------- Custom GCN layer implementation -------------------------
class CustomGCNLayer(nn.Module):
    def __init__(self, in_channels, out_channels, improved=False, cached=False):
        super(CustomGCNLayer, self).__init__()
        self.in_channels = in_channels
        self.out_channels = out_channels
        self.improved = improved
        self.cached = cached
        self.cached_adj = None

        self.weight = nn.Parameter(torch.Tensor(in_channels, out_channels))
        self.bias = nn.Parameter(torch.Tensor(out_channels))
```

```
        self.reset_parameters()

    def reset_parameters(self):
        nn.init.xavier_uniform_(self.weight)
        nn.init.zeros_(self.bias)
        self.cached_adj = None

    def _normalize_adj(self, edge_index, num_nodes, edge_weight=None):
        if edge_weight is None:
            edge_weight = torch.ones(edge_index.size(1), device=edge_index.device)

        # Add self-loops
        self_loop = torch.arange(num_nodes, device=edge_index.device)
        self_loop = torch.stack([self_loop, self_loop], dim=0)
        self_loop_weight = torch.full((num_nodes,), 1.0 if not self.improved else 2.0,
                                      device=edge_index.device)

        # Combine edges and self-loops
        edge_index = torch.cat([edge_index, self_loop], dim=1)
        edge_weight = torch.cat([edge_weight, self_loop_weight], dim=0)

        # Calculate degree matrix
        row, col = edge_index
        deg = torch.zeros(num_nodes, device=edge_index.device)
        deg.scatter_add_(0, row, edge_weight)
        deg_inv_sqrt = deg.pow(-0.5)
        deg_inv_sqrt[deg_inv_sqrt == float('inf')] = 0

        # Normalize adjacency matrix
        edge_weight = deg_inv_sqrt[row] * edge_weight * deg_inv_sqrt[col]

        return edge_index, edge_weight

    def forward(self, x, edge_index, edge_weight=None):
        x = torch.matmul(x, self.weight)

        if self.cached and self.cached_adj is not None:
            edge_index, edge_weight = self.cached_adj
        else:
            edge_index, edge_weight = self._normalize_adj(edge_index, x.size(0), edge_weight)
            if self.cached:
                self.cached_adj = (edge_index, edge_weight)

        row, col = edge_index
```

```
        out = torch.zeros_like(x)
        out.index_add_(0, col, x[row] * edge_weight.unsqueeze(1))
        out += self.bias

        return out


# ------------------------- Custom Transformer implementation -------------------------
class ScaledDotProductAttention(nn.Module):
    def __init__(self, dropout=0.1):
        super(ScaledDotProductAttention, self).__init__()
        self.dropout = nn.Dropout(dropout)

    def forward(self, q, k, v, mask=None):
        d_k = q.size(-1)
        scores = torch.matmul(q, k.transpose(-2, -1)) / np.sqrt(d_k)

        if mask is not None:
            scores = scores.masked_fill(mask == 0, -1e9)

        attn = F.softmax(scores, dim=-1)
        attn = self.dropout(attn)
        output = torch.matmul(attn, v)

        return output, attn


class MultiHeadAttention(nn.Module):
    def __init__(self, d_model, num_heads, dropout=0.1):
        super(MultiHeadAttention, self).__init__()
        assert d_model % num_heads == 0, "d_model must be divisible by num_heads"

        self.d_model = d_model
        self.num_heads = num_heads
        self.d_k = d_model // num_heads

        self.w_q = nn.Linear(d_model, d_model)
        self.w_k = nn.Linear(d_model, d_model)
        self.w_v = nn.Linear(d_model, d_model)
        self.w_o = nn.Linear(d_model, d_model)

        self.attention = ScaledDotProductAttention(dropout)
        self.dropout = nn.Dropout(dropout)
        self.layer_norm = nn.LayerNorm(d_model, eps=1e-6)
```

```
    def forward(self, q, k, v, mask=None):
        batch_size = q.size(0)
        residual = q    # Preserve residual connections

        # Use reshape to ensure memory continuity
        q = self.w_q(q).reshape(batch_size, -1, self.num_heads, self.d_k).transpose(1, 2)
        k = self.w_k(k).reshape(batch_size, -1, self.num_heads, self.d_k).transpose(1, 2)
        v = self.w_v(v).reshape(batch_size, -1, self.num_heads, self.d_k).transpose(1, 2)

        output, attn = self.attention(q, k, v, mask)

        # Use contiguous() to ensure memory continuity
        output = output.transpose(1, 2).contiguous().reshape(batch_size, -1, self.d_model)

        output = self.w_o(output)
        output = self.dropout(output)

        # Use the original input for residual connection
        output += residual

        return self.layer_norm(output), attn


class PositionWiseFeedForward(nn.Module):
    def __init__(self, d_model, d_ff, dropout=0.1):
        super(PositionWiseFeedForward, self).__init__()
        self.fc1 = nn.Linear(d_model, d_ff)
        self.fc2 = nn.Linear(d_ff, d_model)
        self.dropout = nn.Dropout(dropout)
        self.layer_norm = nn.LayerNorm(d_model, eps=1e-6)
        self.activation = nn.GELU()

    def forward(self, x):
        residual = x
        x = self.activation(self.fc1(x))
        x = self.dropout(x)
        x = self.fc2(x)
        x += residual
        return self.layer_norm(x)


class CustomTransformerEncoderLayer(nn.Module):
    def __init__(self, d_model, num_heads, d_ff, dropout=0.1):
```

```
        super(CustomTransformerEncoderLayer, self).__init__()
        self.self_attn = MultiHeadAttention(d_model, num_heads, dropout)
        self.feed_forward = PositionWiseFeedForward(d_model, d_ff, dropout)

    def forward(self, x, mask=None):
        x, attn = self.self_attn(x, x, x, mask)
        x = self.feed_forward(x)
        return x, attn


class CustomTransformer(nn.Module):
    def __init__(self, d_model, num_heads, num_layers, d_ff=2048, dropout=0.1):
        super(CustomTransformer, self).__init__()
        self.layers = nn.ModuleList([
            CustomTransformerEncoderLayer(d_model, num_heads, d_ff, dropout)
            for _ in range(num_layers)
        ])

    def forward(self, x, mask=None):
        attn_list = []
        for layer in self.layers:
            x, attn = layer(x, mask)
            attn_list.append(attn)
        return x, attn_list


# ------------------------- Model definition -------------------------
class GNNModel(nn.Module):
    def __init__(self, num_features, config):
        super(GNNModel, self).__init__()
        self.conv1 = CustomGCNLayer(num_features, config.gcn_hidden_dim1)
        self.conv2 = CustomGCNLayer(config.gcn_hidden_dim1, config.gcn_hidden_dim2)
        self.dropout = nn.Dropout(p=config.dropout_rate)
        self.activation = nn.ReLU()

    def forward(self, data):
        x, edge_index, edge_weight = data.x, data.edge_index, data.edge_attr
        x = self.activation(self.conv1(x, edge_index, edge_weight))
        x = self.dropout(x)
        x = self.conv2(x, edge_index, edge_weight)
        return x


class PositionalEncoding(nn.Module):
```

```
    def __init__(self, d_model, max_len=5000):
        super(PositionalEncoding, self).__init__()
        pe = torch.zeros(max_len, d_model)
        position = torch.arange(0, max_len, dtype=torch.float).unsqueeze(1)
        div_term = torch.exp(torch.arange(0, d_model, 2).float() * (-
torch.log(torch.tensor(10000.0)) / d_model))

        pe[:, 0::2] = torch.sin(position * div_term)
        pe[:, 1::2] = torch.cos(position * div_term)

        self.register_buffer('pe', pe.unsqueeze(0))

    def forward(self, x):
        batch_size, seq_len, _ = x.size()
        pe = self.pe.expand(batch_size, -1, -1)[:, :seq_len, :]
        return x + pe


class TransformerModel(nn.Module):
    def __init__(self, input_dim, output_dim, config):
        super(TransformerModel, self).__init__()
        self.positional_encoding = PositionalEncoding(input_dim)
        self.transformer = CustomTransformer(
            d_model=input_dim,
            num_heads=config.transformer_heads,
            num_layers=config.transformer_layers,
            d_ff=config.transformer_ffn_dim,
            dropout=config.dropout_rate
        )
        self.fc = nn.Linear(input_dim, output_dim)
        self.activation = nn.GELU()

    def forward(self, x):
        x = self.positional_encoding(x)
        x, _ = self.transformer(x)
        x = self.activation(x)
        return self.fc(x.mean(dim=1))


class CombinedModel(nn.Module):
    def __init__(self, gnn_model, transformer_model, num_classes):
        super(CombinedModel, self).__init__()
        self.gnn = gnn_model
        self.transformer = transformer_model
```

```
        self.fc = nn.Linear(
            32 + transformer_model.fc.out_features,
            num_classes
        )
        self.dropout = nn.Dropout(p=0.3)

    def forward(self, data):
        gnn_output = self.gnn(data)

        batch_size = data.num_graphs
        num_nodes_per_graph = gnn_output.size(0) // batch_size
        gnn_output = gnn_output.view(batch_size, num_nodes_per_graph, -1)

        transformer_output = self.transformer(gnn_output)

        gnn_global = gnn_output.mean(dim=1)
        combined = torch.cat((gnn_global, transformer_output), dim=1)
        combined = self.dropout(combined)

        return self.fc(combined)


# ------------------------- Training and evaluation auxiliary function -------------------------
def calculate_accuracy(predicted, true):
    """ Correction: Perform element-level comparison after converting the list to a NumPy array
"""
    predicted_np = np.array(predicted)
    true_np = np.array(true)
    return (predicted_np == true_np).sum() / len(true_np)


def calculate_precision(predicted, true, average='weighted'):
    return precision_score(true, predicted, average=average)


def calculate_recall(predicted, true, average='weighted'):
    return recall_score(true, predicted, average=average)


def calculate_f1(predicted, true, average='weighted'):
    return f1_score(true, predicted, average=average)


def plot_training_curves(train_losses, train_accuracies):
```

```python
    plt.figure(figsize=(12, 5))

    plt.subplot(1, 2, 1)
    plt.plot(train_losses, label="Training Loss")
    plt.title("Training Loss Curve")
    plt.xlabel("Epoch")
    plt.ylabel("Loss")
    plt.legend()
    plt.grid(True, linestyle='--', alpha=0.7)

    plt.subplot(1, 2, 2)
    plt.plot(train_accuracies, label="Training Accuracy", color='orange')
    plt.title("Training Accuracy Curve")
    plt.xlabel("Epoch")
    plt.ylabel("Accuracy")
    plt.legend()
    plt.grid(True, linestyle='--', alpha=0.7)

    plt.tight_layout()
    plt.show()


def plot_confusion_matrix(true_labels, predictions, num_classes=None):
    cm = confusion_matrix(true_labels, predictions)
    plt.figure(figsize=(10, 7))

    # Automatically generate class name labels
    if num_classes is None:
        num_classes = cm.shape[0]
    class_names = [str(i) for i in range(num_classes)]

    sns.heatmap(cm, annot=True, fmt="d", cmap="Blues",
                xticklabels=class_names, yticklabels=class_names)
    plt.title("Confusion Matrix")
    plt.xlabel("Predicted Labels")
    plt.ylabel("True Labels")
    plt.tight_layout()
    plt.show()


def plot_class_distribution(labels, title):
    counter = Counter(labels)
    classes = list(counter.keys())
    counts = list(counter.values())
```

```python
    plt.figure(figsize=(10, 5))
    plt.bar(classes, counts)
    plt.title(title)
    plt.xlabel("Class")
    plt.ylabel("Count")
    plt.xticks(classes, rotation=45)
    plt.tight_layout()
    plt.show()


# ------------------------- Main function for training and evaluation -------------------------
def split_train_test(graphs, config):
    train_size = int(len(graphs) * (1 - config.test_ratio))
    test_size = len(graphs) - train_size
    train_dataset, test_dataset = torch.utils.data.random_split(
        graphs, [train_size, test_size],
        generator=torch.Generator().manual_seed(config.random_state)
    )
    print(f"Train graphs: {len(train_dataset)}, Test graphs: {len(test_dataset)}")

    # Plot class distribution
    train_labels = [graph.y.item() for graph in train_dataset]
    test_labels = [graph.y.item() for graph in test_dataset]

    plot_class_distribution(train_labels, "Training Set Class Distribution")
    plot_class_distribution(test_labels, "Test Set Class Distribution")

    return train_dataset, test_dataset


def get_model_size(model_path):
    try:
        file_size = os.path.getsize(model_path)
        if file_size < 1024:
            return f"{file_size} B"
        elif file_size < 1024 * 1024:
            return f"{file_size / 1024:.2f} KB"
        else:
            return f"{file_size / (1024 * 1024):.2f} MB"
    except:
        return "Unknown"
```

```python
def get_memory_usage():
    process = psutil.Process(os.getpid())
    return process.memory_info().rss / (1024 * 1024)


def train_epoch(model, train_loader, optimizer, criterion, device):
    model.train()
    epoch_loss = 0
    correct = 0
    total = 0

    for batch in train_loader:
        batch = batch.to(device)
        optimizer.zero_grad()

        output = model(batch)
        loss = criterion(output, batch.y.squeeze())

        loss.backward()
        optimizer.step()

        epoch_loss += loss.item()
        _, predicted = torch.max(output.data, 1)

        total += batch.y.size(0)
        correct += (predicted == batch.y.squeeze()).sum().item()

    epoch_loss_avg = epoch_loss / len(train_loader)
    epoch_acc = correct / total

    return epoch_loss_avg, epoch_acc


def evaluate_model(model, test_loader, device):
    model.eval()
    predictions = []
    true_labels = []
    inference_times = []
    max_memory = 0

    initial_memory = get_memory_usage()    # Record initial memory

    with torch.no_grad():
        for batch in test_loader:
```

```
            batch = batch.to(device)
            start_time = time.time()

            current_memory = get_memory_usage()
            output = model(batch)
            current_memory = get_memory_usage()    # Regain memory usage
            max_memory = max(max_memory, current_memory)

            inference_time = time.time() - start_time
            inference_times.append(inference_time)

            _, predicted = torch.max(output.data, 1)
            predictions.extend(predicted.cpu().numpy())
            true_labels.extend(batch.y.squeeze().cpu().numpy())

    return (predictions, true_labels, inference_times, max_memory, initial_memory)


def train_and_evaluate(graphs, num_features, num_classes, model_save_path, config):
    # Device configuration
    device = torch.device('cuda' if torch.cuda.is_available() else 'cpu')
    print(f"Using device: {device}")

    # Data loaders
    train_dataset, test_dataset = split_train_test(graphs, config)
    train_loader = DataLoader(train_dataset, batch_size=config.batch_size, shuffle=True)
    test_loader = DataLoader(test_dataset, batch_size=config.batch_size)

    # Model initialization
    gnn = GNNModel(num_features, config).to(device)
    transformer = TransformerModel(
        input_dim=config.gcn_hidden_dim2,
        output_dim=config.gcn_hidden_dim2,
        config=config
    ).to(device)
    model = CombinedModel(gnn, transformer, num_classes).to(device)

    # Optimizer and loss
    optimizer = optim.Adam(
        model.parameters(),
        lr=config.learning_rate,
        weight_decay=config.weight_decay
    )
    criterion = nn.CrossEntropyLoss()
```

```
    # Learning rate scheduler
    scheduler = optim.lr_scheduler.ReduceLROnPlateau(
        optimizer, 'min', patience=3, factor=0.5, verbose=True
    )

    # Training loop
    train_losses = []
    train_accuracies = []
    best_acc = 0.0

    print("\nStarting training...")
    for epoch in range(config.epochs):
        start_time = time.time()

        epoch_loss, epoch_acc = train_epoch(model, train_loader, optimizer, criterion, device)

        train_losses.append(epoch_loss)
        train_accuracies.append(epoch_acc)

        # Update scheduler
        scheduler.step(epoch_loss)

        end_time = time.time()
        print(f"Epoch {epoch+1}/{config.epochs} - Loss: {epoch_loss:.4f}, Acc:
{epoch_acc:.4f}, Time: {end_time-start_time:.2f}s")

        # Save best model
        if epoch_acc > best_acc:
            best_acc = epoch_acc
            torch.save(model.state_dict(), model_save_path.replace('.pth', '_best.pth'))

    # Plot training curves
    plot_training_curves(train_losses, train_accuracies)

    # Save final model
    torch.save(model.state_dict(), model_save_path)
    print(f"Model saved to {model_save_path} (Size: {get_model_size(model_save_path)})")

    # Load best model for evaluation
    model.load_state_dict(torch.load(model_save_path.replace('.pth', '_best.pth')))

    # Evaluate
    print("\nStarting evaluation...")
```

```
    predictions, true_labels, inference_times, max_memory, initial_memory = evaluate_model(model, test_loader, device)

    # Calculate metrics
    accuracy = calculate_accuracy(predictions, true_labels)
    precision = calculate_precision(predictions, true_labels)
    recall = calculate_recall(predictions, true_labels)
    f1 = calculate_f1(predictions, true_labels)

    # Print metrics
    print(f"\nEvaluation Metrics:")
    print(f"Accuracy: {accuracy:.4f}")
    print(f"Precision: {precision:.4f}")
    print(f"Recall: {recall:.4f}")
    print(f"F1-Score: {f1:.4f}")

    # Detailed classification report
    print("\nClassification Report:")
    print(classification_report(true_labels, predictions))

    # Performance metrics
    avg_inference_time = np.mean(inference_times)
    throughput = len(test_dataset) / sum(inference_times)
    memory_usage = max_memory - initial_memory  # Calculate the actual increased memory

    print(f"\nPerformance Metrics:")
    print(f"Avg inference time: {avg_inference_time:.6f}s")
    print(f"Throughput: {throughput:.2f} samples/sec")
    print(f"Max memory usage: {memory_usage:.2f} MB")

    # Plot confusion matrix
    plot_confusion_matrix(true_labels, predictions, num_classes=num_classes)

    return model


# ------------------------- CICIDS2017 Dataset Processing -------------------------
def run_cicids_experiment(config):
    print("="*70)
    print("                    CICIDS2017 Dataset Experiment                    ")
    print("="*70)

    # Data preprocessing
    if not os.path.exists(config.cicids_processed_path):
```

```
        print("\n1. Preprocessing data...")
        data = load_data(config.cicids_raw_path)
        check_data_quality(data)
        cleaned_data = clean_data(data)
        processed_data, _, _ = process_features(cleaned_data)
        balanced_data = balance_classes(processed_data, config)
        balanced_data.to_csv(config.cicids_processed_path, index=False)
        print(f"Preprocessing completed. Saved to {config.cicids_processed_path}")
    else:
        print(f"\n1. Loading preprocessed data: {config.cicids_processed_path}")
        balanced_data = pd.read_csv(config.cicids_processed_path)

    # Features and labels
    features = balanced_data.iloc[:, :-1].values
    labels = balanced_data.iloc[:, -1].values
    label_encoder = LabelEncoder()
    labels = label_encoder.fit_transform(labels)
    print(f"Features shape: {features.shape}, Classes: {len(np.unique(labels))}")

    # Create graph data
    print("\n2. Creating graph data...")
    graphs = create_graphs(features, labels, config)

    # Train and evaluate
    print("\n3. Training and evaluating model...")
    train_and_evaluate(
        graphs,
        num_features=features.shape[1],
        num_classes=15,
        model_save_path=config.cicids_model_path,
        config=config
    )


# ------------------------- Main function entry -------------------------
def main():
    # Initialize configuration
    config = Config()
    config.print_config()

    # Run experiments
    run_cicids_experiment(config)

    # Clean up
```

```
    gc.collect()
    print("\nAll experiments completed!")


if __name__ == "__main__":
    main()
```
