# Supplementary material for: GT-PNID: A lightweight graph-temporal network integrating GCN and transformer for power network intrusion detection
Source: PLoS One. 2026 Nov 6;21(11):e0360854. doi: 10.1371/journal.pone.0360854 (PMC13742970; doi:10.1371/journal.pone.0360854)
Supplement: Supplementary file 2 [file pone.0360854.s002.pdf]

Supplementary Sensitivity Analysis

1. Sensitivity Analysis for Window Size

The sliding window size significantly impacts model performance. A small window limits the temporal information available in the input sequence, making it difficult for the model to learn complete contextual features. Conversely, an excessively large window increases computational overhead and affects detection timeliness. Based on this trade-off, we conducted a comparative analysis of model performance under different window sizes on both benchmark datasets, as shown in Fig 1.

The experimental results show that as the window size increases from 50 to 80, all evaluation metrics on both datasets exhibit an overall upward trend, reaching optimal levels at 80. When the window size continues to increase beyond 80, model performance declines to varying degrees on both datasets. Further analysis reveals that the performance degradation is more pronounced on the CICIDS2017 dataset, which features more complex attack types and higher feature dimensions. In contrast, the NSL-KDD dataset, with its lower feature dimensionality and more clearly distinguishable attack patterns, exhibits lower sensitivity to window size changes and more stable overall performance. Considering both detection performance and real-time deployment requirements in power system scenarios, the optimal sliding window size is determined to be 80.

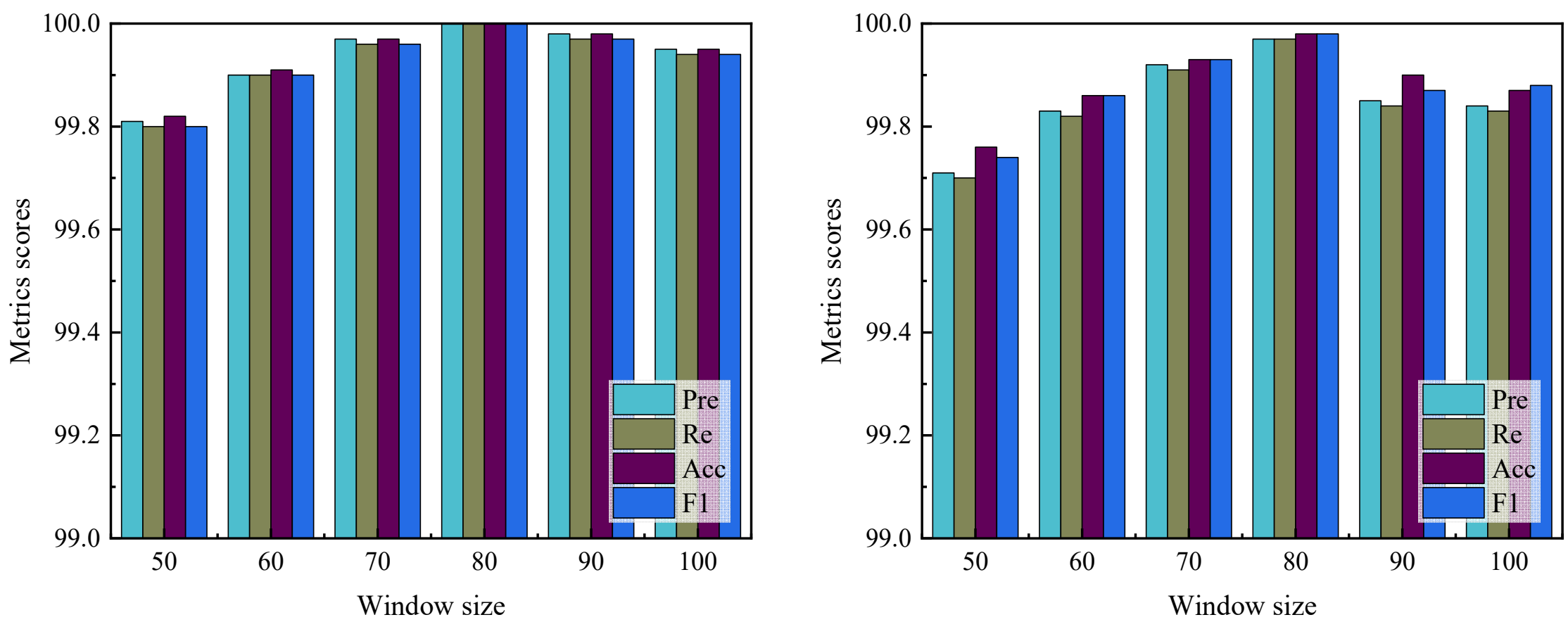

Fig 1. Model performance at different window sizes on (*left*) NSL-KDD and (*right*) CICIDS2017

2. Sensitivity Analysis for Transformer Depth

The number of Transformer encoder layers is a key structural parameter that influences the model's ability to capture temporal dependencies. When the number of layers is too small, the model's capacity to capture long-range temporal correlations is limited, hindering the full exploitation of deep dynamic features within the sequences. Conversely, an excessive number of layers significantly increases parameter count and computational overhead while introducing overfitting risks. To evaluate the impact of encoder depth on detection performance, comparative experiments were conducted on both benchmark datasets. The results are summarized in Tables 1 and 2.

The results show that as the number of encoder layers increases from 2 to 4, detection metrics on both datasets improve significantly, peaking at 4 layers, which indicates that spatio-temporal features

achieve deep coupling under this configuration. Further increasing the number of layers leads to continuous performance degradation. The CICIDS2017 dataset, with its more complex traffic temporal patterns, exhibits a stronger tendency to overfit, leading to more severe performance degradation. Balancing detection performance with lightweight deployment requirements, the optimal number of encoder layers is determined to be 4.

Table 1. Model performance at different Transformer encoder depths on the NSL-KDD dataset.

Number of Encoder Layers	Pre (%)	Re (%)	Acc (%)	F1 (%)
2	99.55	99.54	99.56	99.54
4	100	100	100	100
6	99.97	99.96	99.97	99.96
8	99.91	99.9	99.92	99.9

Table 2. Model performance at different Transformer encoder depths on the CICIDS2017 dataset.

Number of Encoder Layers	Pre (%)	Re (%)	Acc (%)	F1 (%)
2	99.4	99.38	99.42	99.39
4	99.97	99.97	99.98	99.98
6	99.92	99.91	99.93	99.91
8	99.86	99.84	99.87	99.85

3. Sensitivity Analysis for Initial Learning Rate

With the model architecture fixed, the learning rate is a core optimization hyperparameter that controls the parameter update step size and governs training convergence. An excessively large learning rate causes training instability and prevents convergence, while an excessively small learning rate results in slow convergence and susceptibility to local optima. Therefore, we conducted systematic experiments with learning rates of different magnitudes on the benchmark datasets. The corresponding performance statistics are presented in Tables 3 and 4.

The results show that on both datasets, model performance improves substantially as the learning rate decreases from 0.1 to 0.0001. Under large learning rates, severe training oscillations occur and performance is suboptimal, with the high-dimensional and complex CICIDS2017 dataset being more severely affected. At a learning rate of 0.0001, the training process stabilizes and all detection metrics reach optimal values simultaneously on both benchmark datasets, demonstrating good convergence and generalization capability. Balancing convergence stability and final detection performance, the optimal initial learning rate is determined to be 0.0001.

Table 3. Model performance at different initial learning rates on the NSL-KDD dataset.

Initial Learning Rate	Pre (%)	Re (%)	Acc (%)	F1 (%)
0.1	93.15	92.37	93.42	92.76
0.01	97.08	96.94	97.25	97.01
0.001	99.07	99.02	99.14	99.04
0.0001	100	100	100	100

Table 4. Model performance at different initial learning rates on the CICIDS2017 dataset.

Initial Learning Rate	Pre (%)	Re (%)	Acc (%)	F1 (%)
0.1	91.87	90.62	92.15	91.24
0.01	96.12	95.87	96.38	95.99
0.001	98.54	98.42	98.67	98.48
0.0001	99.97	99.97	99.98	99.98
