# Supplementary material for: GT-PNID: A lightweight graph-temporal network integrating GCN and transformer for power network intrusion detection
Source: PLoS One. 2026 Nov 6;21(11):e0360854. doi: 10.1371/journal.pone.0360854 (PMC13742970; doi:10.1371/journal.pone.0360854)
Supplement: Supplementary file 3 [file pone.0360854.s003.pdf]

Window Purity Analysis

A detailed label ambiguity analysis is presented for the more complex CICIDS2017 dataset, which comprises 15 fine-grained classes and more heavily interleaved traffic patterns; by contrast, NSL-KDD contains only 5 coarse-grained classes, whose attack traffic typically manifests as long, continuous bursts.

The window purity analysis is conducted on the preprocessed CICIDS2017 sequence (via Algorithm 2), with the training set containing 1,585,017 samples partitioned into 39,624 windows, the validation set 317,003 samples into 7,924 windows, and the test set 396,254 samples into 9,905 windows. Full results are summarized in Table S3.

Table S3 Window composition and purity statistics for the preprocessed CICIDS2017 sequence (ω = 80, δ = 40)

Set	Number of windows	Mixed-label (%)	Purity=100% (%)	Purity>95% (%)	Normal majority but attack label (%)
Train	39,624	5.8	94.2	98.1	0.8
Validation	7,924	4.9	95.1	98.3	0.7
Test	9,905	4.6	95.4	98.2	0.6

Note:

- Mixed-label windows contain >=2 distinct sample labels. For the test set, 4.6% mixed-label test windows can be further decomposed into two subgroups: attack-majority mixed windows (~4.0% of the test set), normal-majority mixed windows labeled as attack (0.6% of the test set).
- Purity is defined as the fraction of samples in a window sharing the dominant label, $\text{Purity} = \frac{\max_{c \in \mathbf{c}_u} \text{count}(c)}{\omega}$, where c_u is the graph-level label, $\mathbf{c}_u = [c^{(1)}, c^{(2)}, \ldots, c^{(\omega)}]$ denotes the set of sample labels within a window, and ω is the window size.
- The "Purity > 95%" metric includes all 100%-pure windows.
- A "normal majority but attack label" window is defined as a window containing at least one attack sample, where normal samples constitute >50% of the window (i.e., normal samples outnumber attack samples), and the window is labeled as attack per our revised labeling rule.
