# Supplementary material for: GT-PNID: A lightweight graph-temporal network integrating GCN and transformer for power network intrusion detection
Source: PLoS One. 2026 Nov 6;21(11):e0360854. doi: 10.1371/journal.pone.0360854 (PMC13742970; doi:10.1371/journal.pone.0360854)
Supplement: Supplementary file 4 [file pone.0360854.s004.pdf]

The following table summarizes the time complexity order, estimated FLOPs, and proportion of each module.

Time Complexity Breakdown

Module	Complexity Order	FLOPs	Proportion
Dynamic Graph Construction	$O(\omega^2 \cdot \rho)$	5.0×10^5	5.4%
GCN encoder	$O(\omega \cdot k \cdot d_g^2)$	8.2×10^5	8.9%
Transformer encoder	$O(\mathcal{L}_u \cdot \omega \cdot d_u(\omega + d_{\text{FFN}}))$	7.8×10^6	85.6%
Classification	$O\left((d_g + d_u) \cdot d_{\text{FCN}}\right)$	1.0×10^4	0.1%
Total	—	$\mathbf{9.13 \times 10^6}$	100%

**Symbol**	**value**	**Interpretation**
ω	80	Sliding window size
ρ	78	Node feature dim
k	10	Top-*k* neighbors
d_g	32	GCN hidden dim
$\mathcal{L}_u$	4	Number of layers in the Transformer encoder
d_u	32	Transformer hidden dim
d_{FFN}	128	FNN hidden dim
d_{FCN}	128	FCN hidden dim
