# Supplementary material for: GT-PNID: A lightweight graph-temporal network integrating GCN and transformer for power network intrusion detection
Source: PLoS One. 2026 Nov 6;21(11):e0360854. doi: 10.1371/journal.pone.0360854 (PMC13742970; doi:10.1371/journal.pone.0360854)
Supplement: Supplementary file 5 [file pone.0360854.s005.pdf]

Log Dimension	Parameter	PyTorch (FP32) Mode	TensorRT (FP16) Accelerated Mode
Basic Configuration	Total Independent Inference Runs	1000	1000
	Test Dataset	CICIDS2017 Preprocessed Set	CICIDS2017 Preprocessed Set
	Samples per Inference	1 Generated Dynamic Graph Sample	1 Generated Dynamic Graph Sample
Inference Latency	Average Inference Latency (ms)	3.21	1.68
	Minimum Inference Latency (ms)	2.97	1.42
	Maximum Inference Latency (ms)	3.68	1.93
	Latency Standard Deviation (ms)	0.11	0.08
Resource Utilization	Average GPU Memory Usage (MB)	168	110
	Peak GPU Memory Usage (MB)	179	117
	Average CPU Utilization (%)	22.7	14.3
Accuracy Performance	Overall Classification Accuracy (%)	99.98	99.97
	Average Precision per Class (%)	99.97	99.96
	Average Recall per Class (%)	99.97	99.96
	Average F1-Score per Class (%)	99.97	99.96
